# Comparative genomic and transcriptomic analyses of trans-kingdom pathogen *Fusarium solani* species complex reveal degrees of compartmentalization

**DOI:** 10.1186/s12915-022-01436-7

**Published:** 2022-10-20

**Authors:** Daphne Z. Hoh, Hsin-Han Lee, Naohisa Wada, Wei-An Liu, Min R. Lu, Cheng-Kuo Lai, Huei-Mien Ke, Pei-Feng Sun, Sen-Lin Tang, Wen-Hsin Chung, Ying-Lien Chen, Chia-Lin Chung, Isheng Jason Tsai

**Affiliations:** 1 Biodiversity Research Center, Academia Sinica, 115 Nangang, Taipei, Taiwan; 2 Biodiversity Program, Taiwan International Graduate Program, Academia Sinica and National Taiwan Normal University, Taipei, Taiwan; 3 Department of Life Science, National Taiwan Normal University, 116 Wenshan, Taipei, Taiwan; 4 Genome and Systems Biology Degree Program, National Taiwan University and Academia Sinica, Taipei, Taiwan; 5 Department of Plant Pathology, National Chung Hsing University, Taichung, Taiwan; 6 Department of Plant Pathology and Microbiology, National Taiwan University, Taipei, 10617 Taiwan

**Keywords:** Genome compartments, Fusarium solani species complex, Chromosome evolution, Opportunistic pathogen, Animal pathogenicity, Turtle

## Abstract

**Background:**

The *Fusarium solani* species complex (FSSC) comprises fungal pathogens responsible for mortality in a diverse range of animals and plants, but their genome diversity and transcriptome responses in animal pathogenicity remain to be elucidated. We sequenced, assembled and annotated six chromosome-level FSSC clade 3 genomes of aquatic animal and plant host origins. We established a pathosystem and investigated the expression data of *F. falciforme* and *F. keratoplasticum* in Chinese softshell turtle (*Pelodiscus sinensis)* host.

**Results:**

Comparative analyses between the FSSC genomes revealed a spectrum of conservation patterns in chromosomes categorised into three compartments: core, fast-core (FC), and lineage-specific (LS). LS chromosomes contribute to variations in genomes size, with up to 42.2% of variations between *F. vanettenii* strains. Each chromosome compartment varied in structural architectures, with FC and LS chromosomes contain higher proportions of repetitive elements with genes enriched in functions related to pathogenicity and niche expansion. We identified differences in both selection in the coding sequences and DNA methylation levels between genome features and chromosome compartments which suggest a multi-speed evolution that can be traced back to the last common ancestor of *Fusarium*. We further demonstrated that *F. falciforme* and *F. keratoplasticum* are opportunistic pathogens by inoculating *P. sinensis* eggs and identified differentially expressed genes also associated with plant pathogenicity. These included the most upregulated genes encoding the CFEM (Common in Fungal Extracellular Membrane) domain.

**Conclusions:**

The high-quality genome assemblies provided new insights into the evolution of FSSC chromosomes, which also serve as a resource for studies of fungal genome evolution and pathogenesis. This study also establishes an animal model for fungal pathogens of trans-kingdom hosts.

**Supplementary Information:**

The online version contains supplementary material available at 10.1186/s12915-022-01436-7.

## Background

The filamentous fungi in the genus *Fusarium* are amongst the most virulent pathogens affecting multi-kingdom hosts [1, 2], but can also exist as saprophytes. The genus, commonly identified as plant pathogens, has caused devastating losses in the global agricultural industry [3] and is recognised as one of the most prevalent clinical pathogens causing superficial and invasive disease in immunocompromised humans [4, 5]. In recent decades, an increasing number of fusariosis cases associated with various types of animals have been reported worldwide [6–10], but research on the virulence of pathogens causing fusariosis beyond plant hosts remains limited [11, 12]. The disease is now considered to be a serious emerging fungal threat potentially inducing host population loss and extinction [2, 13].

The most prevalent *Fusarium* pathogens associated with veterinary infection are from the species-rich clade three of *Fusarium solani* species complex (FSSC) [1, 2, 14, 15]. Most species in this clade are ubiquitous in the natural environment [1], except *F. keratoplasticum*, which is mostly isolated from plumbing systems [16]. Several FSSC species were reported to cause disease in aquatic animals such as grey seals, shrimps, dolphins, and sharks [2]. Two species from the FSSC clade three—*F. falciforme* and *F. keratoplasticum*—were the predominant species occurring in diseased sea turtle nests worldwide [17], and the latter was also recently reported to infect *Podocnemis unifilis*, an endangered freshwater turtle species [18]. Koch’s postulates were fulfilled for *F. keratoplasticum*, which can cause disease and high mortality rate (83.3%) in sea turtle *Caretta caretta* eggs [19]. The disease is now termed sea turtle egg fusariosis [20] and is responsible for low hatching success of eggs from both natural nests [17, 21] and man-made hatcheries [22, 23].

One particular genome characteristic in many pathogenic fungi are that their chromosomes can be differentiated into two compartments: the core chromosome (CC), which contain essential genes required for survival and reproduction, and lineage-specific chromosome (LSC; also known as accessory chromosome), which is repeat-rich and enriched with genes mostly associated with niche adaptation and pathogenicity [24–26]. LSC can be dispensable and do not affect fungal growth in several *Fusarium* species such as *F. oxysporum* f. sp. *lycopercisi* [24] and *F. vanettenii* (previously *Nectria haematococca*) [27]. The LSC of *F. vanettenii* harbours pea-specific pathogenic genes—the PEP cluster [27–29], and the deletion of the entire LSC reduced the pathogen’s virulence towards pea plants [11, 27].

In this study, we produced six high-quality genome assemblies for species in the FSSC clade 3 isolated from hosts belonging to different kingdoms. Comparative analyses of six FSSC and three other *Fusarium* genomes allowed us to divide the chromosomes into multiple compartments based on genome alignments and clustering of single-copy orthologs. These compartments harboured distinct genome features as a result of different evolutionary dynamics. We show that *F. falciforme* and *F. keratoplasticum* that are responsible for mass mortalities in sea turtles can penetrate Chinese softshell turtle eggshells and colonise egg inclusions in the laboratory setting. By employing a dual RNA-seq approach, gene expression data provided a detailed description of the two species and the host during infection. Together, the availability of these genomes and the transcriptome data underpin an initial effort to study animal parasitism in these trans-kingdom pathogens.

## Results

### Genome characteristics of six sequenced FSSC isolates

We sequenced six genomes of five species within clade three of FSSC (Table 1). This included *F. falciforme* (Fu3), *F. keratoplasticum* (Fu6 and LHS11), and *Fusarium* sp. FSSC12 (LHS14) isolated from various aquatic animal hosts. Two species of plant host origins—*Fusarium* sp. (Ph1) from orchid and *F. vanettenii* (Fs6) from pea—were also chosen for comparative purposes. The initial assemblies were produced from averaging 121X of Oxford Nanopore reads (sequence N50 = 14–23kb) using the Flye assembler [30] and polished by Illumina reads (Additional file 1: Table S1). The final assemblies were in 14 to 40 contigs with N50 3.2–4.2Mb (Table 1) averaging 56 Mb, which is more contiguous and larger than other representative *Fusarium* genomes (ranging 12–4197 contigs averaging 47Mb; Additional file 1: Table S2). Interestingly, *F. vanettenii* Fs6 has an assembly of 72.9Mb, the largest *Fusarium* genome reported to date and 42.2% larger than the published *F. vanettenii* MPVI 77-13-4 (abbreviated as FVANE) genome of 51.2Mb [31], suggesting high intraspecies variation. The *F. falciforme* Fu3 reference was amongst the most complete genome, consisting of 14 contigs with six telomere-to-telomere gapless chromosomes (Additional file 1: Table S3) and was on average 17 times more contiguous than the published FSSC genomes (N90: 2.7Mb vs. 0.4kb–2.6Mb, respectively; last accessed date 18th January 2022; Additional file 1: Table S4).

**Table 1:** Genome statistics of six sequenced isolates of *Fusarium solani* species complex

Species	*F. falciforme*	*F. keratoplasticum*	*F. keratoplasticum*	*Fusarium sp.* FSSC12	*Fusarium sp.*	*F. vanettenii*
**Strain ID**	Fu3	Fu6	LHS11	LHS14	Ph1	Fs6
**Estimated haploid genome size (Mb)**	54.5	51.6	56.2	59.6	50.9	72.9
**Assembly size (Mb)**	53.6	49.5	53.5	56.3	51.5	72.9
**Number of contig**	14	25	26	35	24	40
**Largest contig (Mb)**	6.8	6.4	6.6	6.1	5.4	6.7
**N50 Mb (L50)**	3.8 (6)	4.2 (5)	3.4 (6)	3.8 (6)	3.2 (6)	3.3 (9)
**N90 Mb (L90)**	2.7 (12)	2.2 (11)	1.5 (13)	0.9 (14)	1.1 (17)	1.3 (23)
**GC %**	49.4	51.3	51.4	48.6	51.1	49.4
**Number of protein coding genes**	15137	14935	15051	15937	15498	18862
**Protein BUSCO % (fungi)**	99	98.4	98.4	98.3	100	98.6
**Protein BUSCO % (ascomycota)**	98.8	98.8	98.1	98.6	99.8	98.7

Genome size was estimated via GenomeScope 2.0

We predicted 14,927 to 18,862 protein-encoding genes from these assemblies using the MAKER2 [32] pipeline based on evidence using proteomes from closely related fungi and transcriptome reads generated from mycelium. Benchmarking Universal Single-Copy Orthologs (BUSCO) [33] analysis indicated that the proteomes were 98.3 to 100% complete (Table 1) and 70–77.1% of these genes were assigned to a protein family or domain (Pfam) via eggNOG-mapper [34], indicating high completeness in these assemblies. The orthology of the six sequenced assemblies, 17 other *Fusarium* genomes, and an outgroup species *Beauveria bassiana* (Additional file 1: Table S2) was inferred using OrthoFinder [35]. A total of 24,203 orthogroups (OGs) were inferred, of which 16,154 (66.7%) and 235 (1%) OGs were *Fusarium* and FSSC-specific, respectively. All six genomes contained a predicted average of 420 (2.7% of total gene) and 487 genes, encoding effectors and carbohydrate-active enzymes (CAZYmes), respectively (Additional file 1: Table S5 and S6). In addition, an average of 44 secondary metabolite biosynthetic gene clusters (SMBGCs) were detected in the six genomes, some of which, including a fusarin gene cluster, are associated with plant pathogenicity (Additional file 1: Table S7 and S8) [36]. The numbers of these gene families are comparable to other *Fusarium* genomes [36, 37]. The repeat proportion of FSSC genomes typically range 4.2–9.4%, except for *F. vanettenii* Fs6, which contained 18.8% repeats (Additional file 2: Fig. S1a). DNA transposons constituted the largest proportion (1.4 to 6.4%) of FSSC genomes (Additional file 2: Fig. S1b), followed by LTR retrotransposons (0.9 to 5.8%).

**Fig. 1:**
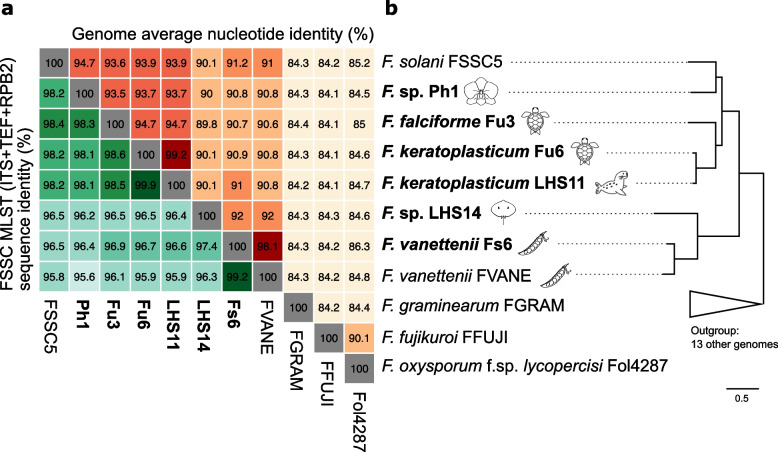
Average nucleotide similarities and species tree of *Fusarium solani* species complex (FSSC). **a** Nucleotide identities determined by multi-locus sequences (ITS+TEF+RPB2) commonly used for FSSC in the lower green-shaded triangular matrix and genome average nucleotide identity in selected *Fusarium* species in the upper orange-shaded triangular matrix. Darker shading indicates higher sequence similarity. **b** A simplified *Fusarium* species tree with outgroup species collapsed. The full phylogeny is in Additional file 2: Fig. S2 and constructed using 2385 single-copy orthogroup sequences. Species name in bold represents the strains sequenced in current study and source origin (host) represented by icons

To determine the phylogenetic position of our isolates, we compared the sequence identities and constructed a maximum likelihood phylogeny based on 40 FSSC species using the commonly employed multi-locus sequence typing (MLST) targeting the internal transcribed spacer (ITS) region, translation elongation factor (TEF), and RNA polymerase II (RPB2) (Fig. 1a; Additional file 2: Fig. S2 and Additional file 1: Table S9). With the new genomes, we computed the genome average nucleotide identity (ANI) and constructed a species phylogeny using 2385 single-copy orthologs, which recapitulated the relationship of the MLST phylogeny, and found that the species relationships were not grouped by animal or plant hosts (Fig. 1b; Additional file 2: Fig. S3 and Additional file 1: Table S2). Ph1 was most closely related to *F. solani* haplotype FSSC5 (MLST and genome ANI: 98.2 and 94.7%). *Fusarium* sp. LHS14 was designated as haplotype FSSC12 and the first genome assembly of this species (Additional file 2: Fig. S3).

### Evolutionary dynamics of FSSC chromosomes

**Fig. 2:**
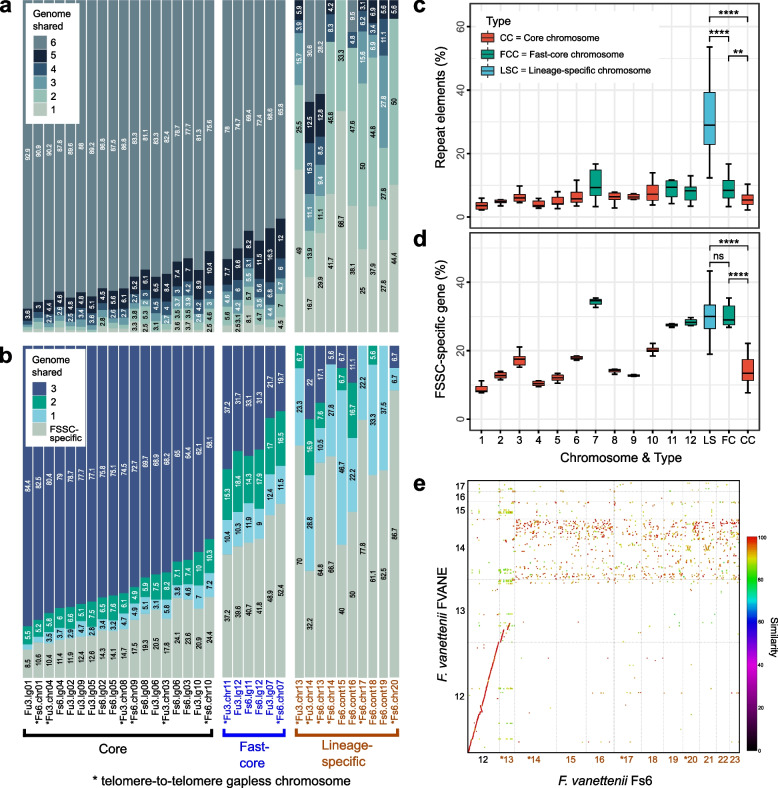
Shared ortholog and structural features of *Fusarium solani* species complex chromosomes. **a** Proportion of one-to-one ortholog shared across six genomes. Figure shows only chromosome of *F. falciforme* Fu3 and *F. vanettenii* Fs6. See Additional file 2: Fig. S4 for all six genomes. **b** Proportion of one-to-one ortholog shared with three additional *Fusarium* genomes outside of FSSC including *F. oxysporum*, *F. graminearum*, and *F. fujikuroi*. **c** Proportions of repeat elements and **d** FSSC-specific gene of each chromosome and chromosome type. Statistical significance was calculated using Wilcoxon rank sum test (**: *p* < 0.01; ****: *p* < 0.0001; ns: *p* > 0.05). **e** Subset of synteny dotplot between *F. vanettenii* Fs6 lineage-specific chromosomes (numbered in colour brown) and FVANE. Full genome dotplot can be found in Additional file 2: Fig. S6

To investigate whether lineage-specific chromosomes (LSCs) were present in each *Fusarium* species, we assessed the extent of chromosome linkage between species via pairwise single-copy orthologs, proportions of repetitive elements and FSSC-specific genes in each chromosome. In addition to assigning the core and LSC as in previous *Fusarium* studies [24, 31], FSSC chromosomes were categorised into two compartments, revealing a spectrum of conservation (Fig. 2a; Additional file 2: Fig. S4a). The majority of chromosomes were part of the linkage group that was shared by all six species; we designated these the core chromosomes (CCs; Additional file 2: Fig. S4a). On the other end of the conservation spectrum were LSCs that contained single-copy orthologues shared by mostly two out of six genomes and a lower proportion of shared genes. Further examination of linkage between FSSC chromosome with three other non-FSSC *Fusarium* species revealed an additional compartment within the FSSC, which had a lower proportion of genes with orthology detected beyond FSSC (Fig. 2b; Additional file 2: Fig. S4b). This additional compartment comprised mainly three linkage groups corresponding to chromosomes 7, 11, and 12 of *F. falciforme* Fu3 genome; these were designated as fast-core chromosomes (FCCs).

Synteny analysis using *F. falciforme* Fu3 as reference revealed that the CCs and FCCs were more syntenic than the LSCs between the FSSC species (Additional file 2: Fig. S5). The CCs harboured significantly fewer repeats (Wilcoxon rank sum test, *p* < 0.01; Fig. 2c) and lower proportions of FSSC-specific genes than FCCs and LSCs in each FSSC genome (Wilcoxon rank sum test, *p* < 0.0001; Fig. 2d). We designated a total of 9 CCs, 3 FCCs, and 2–11 LSCs in each FSSC genome. The CCs and FCCs constituted the majority (67.7–94.7%) of genomes, while the number of LS chromosomes varied and were responsible for the aforementioned genome differences between the two *F. vanettenii* strains (72.9Mb in Fs6 versus 51.2Mb in FVANE). These LS chromosomes exhibited a lack of synteny (Fig. 2e; Additional file 2: Fig. S6), suggesting that these chromosomes were differentially maintained or distributed through horizontal chromosome transfer [24]. Additionally, the *F. vanettenii* CCs comprise 14,513 and 14,961 genes in FVANE and Fs6, respectively, but the latter contained more genes (3395 vs. 1195) located on these additional LSCs. The gapless FSSC LS chromosomes averaged 1.2Mb, which is a similar mark up as the *F. oxysporum* genome [24]. However, telomere-to-telomere LSC length were observed in FSSCs, ranging from 0.8Mb in chromosome 13 of *Fusarium* sp. Ph1 to 3.3Mb in chromosome 14 of *F. vanettenii* Fs6, demonstrating the characteristic dynamics of non-CCs in *Fusarium* genomes [38, 39]. The combined mean size of FCCs was approximately 2.8Mb, with FSSC-specific genes ranging from 26.8 to 35.4% of the total gene content (vs. 7.7 to 22.1% in CCs). A lower number of FSSC-specific genes were detected in LSCs compared to CCs (averaging 59 vs. 272 genes), but the former constitutes at least one-fifth of the total gene content (Additional file 1: Table S10). The FCCs appeared as an intermediate category between the CCs and LSCs based on genome features, similar to *F. oxysporum* [38]. In terms of gene locality, sub-telomeric bias was detected for these FSSC-specific genes, a similar trend as previously reported in *F. graminearum* [40] and *Aspergillus* genomes [41], but not in FCCs, where the genes were distributed across the entire chromosome (Additional file 2: Fig. S7). Gene Ontology (GO) enrichment analysis in each chromosome type revealed FCCs and LSCs harbour genes that are feasibly linked to pathogenicity and expansion of new niches such as environment or host, compared to CCs which mainly harbour genes for essential biological functions such as growth and development (Additional file 3). FCCs had the highest mean proportion and number of effectors, CAZYmes and SMBGCs amongst the chromosome types (Additional file 1: Table S11 and S12), consistent with observations in *F. oxysporum* [42]*.* Together these results suggest that, in addition to LSCs, genes in FCCs also play an important role in pathogenicity processes such as host colonisation and infection.

**Fig. 3:**
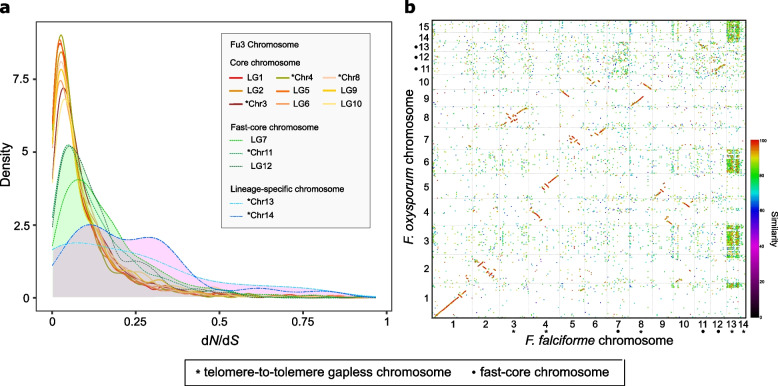
Evolutionary dynamics and origin of fast-core chromosomes. **a** The density of *d*_N_/*d*_S_ in *F. falciforme* Fu3 chromosomes for each single-copy ortholog gene paired with *F. keratoplasticum* Fu6. **b** Syntenic dotplot produced via PROmer comparing between *F. falciforme* Fu3 and *F. oxysporum* f. sp. *lycopercisi* 4287 genomes

To investigate the evolutionary differences of each chromosome type, we estimated the ratio of non-synonymous substitutions to synonymous substitutions per gene (*d*_N_/*d*_S_) across single-copy orthologs between *F. falciforme* Fu3 and *F. keratoplasticum* Fu6. Overall, genes located in LSCs had higher *d*_N_/*d*_S_ than FCCs, followed by CCs (median *ω* = 0.24, 0.10, and 0.06, respectively, Wilcoxon rank sum test, *p* < 0.05 in all comparison pairs; Fig. 3a), indicating different levels of purifying selections amongst chromosome types. In addition, genes that were translocated to other linkage groups were also comprised of higher *d*_N_/*d*_S_ (median *ω* = 0.09, 0.08, and 0.05, in LSCs, FCCs, and CCs, respectively; Wilcoxon rank sum test, *p* < 0.0001 in all comparison pairs; Additional file 2: Fig. S8), suggesting differential selection after rearrangement, such as relaxed purifying selection.

Several distinctive differences in chromosome 7 of FSSC genomes were observed when compared to the other FCCs. For instance, the former was mostly located at the end of FCCs in the conservation spectrum (Additional file 2: Fig. S4); it had the highest proportions of repeat and FSSC-specific genes (Fig. 2c, d) and higher *d*_N_/*d*_S_ ratio than other FCCs and CCs (Fig. 3a). Furthermore, frequent rearrangements were observed in chromosome 7 across FSSC genomes (Additional file 2: Fig. S5). Interestingly, intraspecies chromosomal structural variations were observed between *F. keratoplasticum* Fu6 and LHS11 in which translocations of *F. falciforme* Fu3 chromosome 7 were observed in chromosomes 3, 13, and 14 of *F. keratoplasticum* Fu6 but dissimilar in LHS11 (Additional file 2: Fig. S5). Hence, the shorter length of chromosome 7 of Fu6 suggested that accessory regions were lost and translocated to other chromosomes in the genome. Enriched clustering of single-copy orthologs and synteny were found between FCCs of the *F. falciforme* Fu3 and *F. oxysporum* f. sp. *lycopercisi* strain 4287 genomes (Fig. 3b; Additional file 2: Fig. S9), but not other *Fusarium* species (Additional file 2: Fig. S10), suggesting the FCCs in the FSSCs were present in the last common ancestor of *Fusarium* species and were differentially maintained.

### Interplay between DNA methylation and repeat in different compartments

**Fig. 4:**
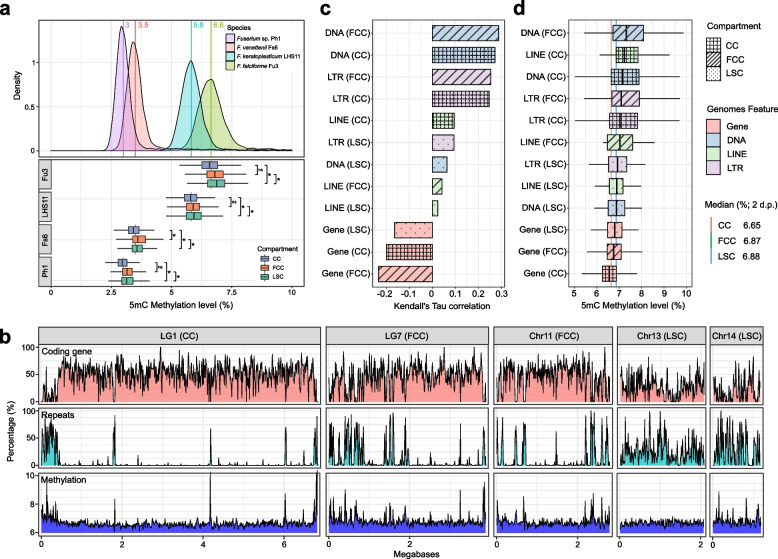
5mC DNA methylation levels in *Fusarium solani* species complex genomes and its chromosome types. **a** Methylation density and level of each genome and the chromosome types. Number and line indicate genome’s methylation median. Statistical significance was calculated using Wilcoxon rank sum test (*: *p* < 0.0001). **b** Percentages of coding gene, repeats, and methylation across 10-kb window of *F. falciforme* Fu3 chromosomes. **c** Pearson correlation coefficient between proportion of genome features and methylation level in *F. falciforme* Fu3. **d** Methylation level of genome features in *F. falciforme* Fu3. CC, FCC, and LSC represents core chromosome, fast-core chromosome, and lineage-specific chromosome, respectively

Examination of 5-methylcytosine (5mC) in the DNA of four of our FSSC genomes revealed that methylation levels were different between species ranging from 3 to 6.6% (Fig. 4a). The distinct differences were not associated to phylogenetic positions, but higher methylation abundance was observed in the strains isolated from animals (Fig. 1b). Significantly higher DNA methylation levels were observed in FCCs and LSCs than in CCs (Wilcoxon rank sum test, *p* < 0.0001; Fig. 4a; Additional file 2: Fig. S11). Consistent with other fungi [43, 44], the 5mC methylation level in the FSSCs were typically higher in the repeat regions than in coding regions (Fig. 4b; Additional file 2: Fig. S12a to S14a). As expected, we found methylation levels along the genome were negatively and positively correlated with the presence of coding sequence and repeat region, respectively (Kendall’s Tau correlation, *p* < 0.0001 in all four species; Fig. 4c; Additional file 2: Fig. S12b to S14b). Strikingly, we found uniformly low methylation levels across LSCs (example with chr. 13 and 14 in Fig. 4b; Additional file 2: Fig. S12a and S13a). In contrast to CCs and FCCs, methylation levels were similar between the repetitive elements and coding regions in LSCs (Tukey HSD test; Fig. 4d; Additional file 1: Table S13; Additional file 2: Fig. S12c to S14c), which were also consistent with the lower correlation between methylation level and genome features (Fig. 4c; Additional file 2: Fig. S12b to S14b). Together, the overall different methylation levels between the CCs and FCCs were due to the different markup of coding and repeat types (Fig. 2c; Additional file 2: Fig. S15). Despite higher repeat content in the LSCs, DNA methylation levels were uniform and overall similar to the FCCs.

### *Fusarium falciforme* and *F. keratoplasticum* are opportunistic pathogens of turtle eggs

**Fig. 5:**
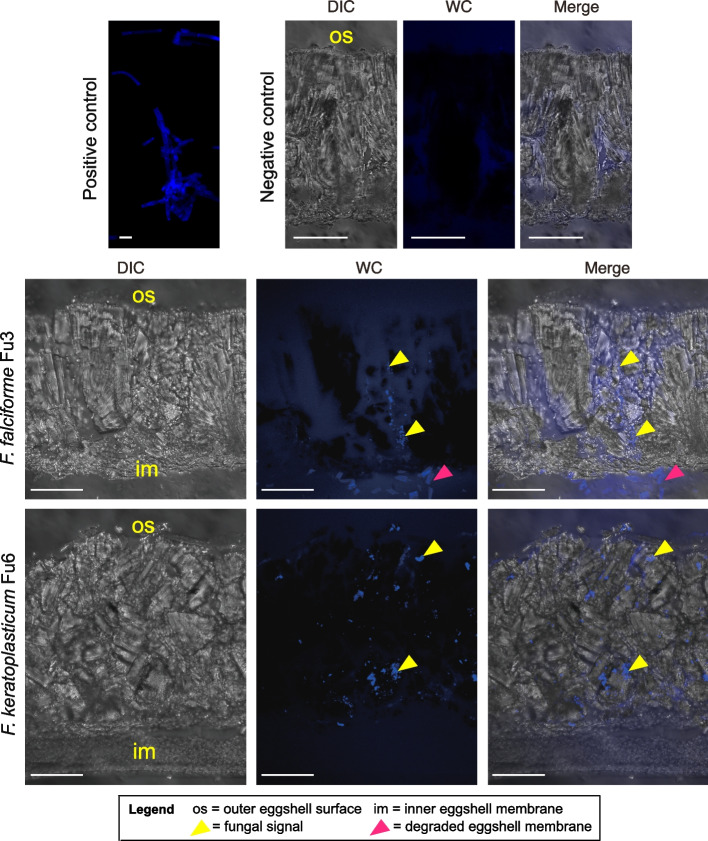
Laser confocal microscopy images of *Pelodiscus sinensis* eggshell cross section inoculated by *Fusarium solani* species complex. The eggshell was undecalcified and acquired at 5-day post-infection of *F. falciforme* Fu3 and *F. keratoplasticum* Fu6. Fungal material was stained with Calcofluor White (blue signal). DIC = differential interference contrast. WC = white contrast. Scale bar in figures is 50μm except 10μm in positive control

*F. falciforme* Fu3 and *F. keratoplasticum* Fu6 are frequently isolated from sea turtle eggs [17, 22], and we seek to understand how these two species colonise and invade egg hosts by inoculating Chinese softshell turtle (*Pelodiscus sinensis*) eggs. These two *Fusarium spp.* did not actively seek hosts because attraction assays revealed no significant difference in hyphal growth rates with or without the presence of turtle eggs in each *Fusarium* species (Additional file 2: Fig. S16; Wilcoxon test, *p* = 0.67 in *F. falciforme* and *p* = 0.86 in *F. keratoplasticum*) or between species (Wilcoxon test, *p* = 0.86 in control and *p* = 0.86 in treatment). After 5 days of inoculation, hyphal growth in both species was observed on the eggshell surface (Fig. 5) with some occasionally growing into the cavity-like structures (Additional file 2: Fig. S17). Cryosectioning and histological observations of undecalcified eggshell cross sections (see Additional file 3 for sample preparations) [45] revealed the presence of both *Fusarium* species on the outer, within the calcareous, and inner layers of the eggshells, confirming that hyphae vertically penetrated the eggshell. Degradations were sometimes observed on the eggshell membrane (Fig. 5). We further examined the symptoms of *F. falciforme* and *F. keratoplasticum* colonisation on eggs at 3 and 4 days post-inoculation (dpi) (Additional file 2: Fig. S18). Mycelial mass was observed growing on the membrane (Additional file 2: Fig. S18a) and embryo (Additional file 2: Fig. S18b and c). Some inoculated eggs exhibited reduced branching points and disrupted blood capillaries on the microvascular system despite the embryo still being alive during the examination (Additional file 2: Fig. S18c). Together, these observations suggested that these two FSSC species are opportunistic animal pathogens and, upon contact, their hyphae can penetrate eggshells via natural openings and subsequently lyse and colonise egg inclusions.

### Transcriptome profiles of FSSC pathogens during egg infection

**Fig. 6:**
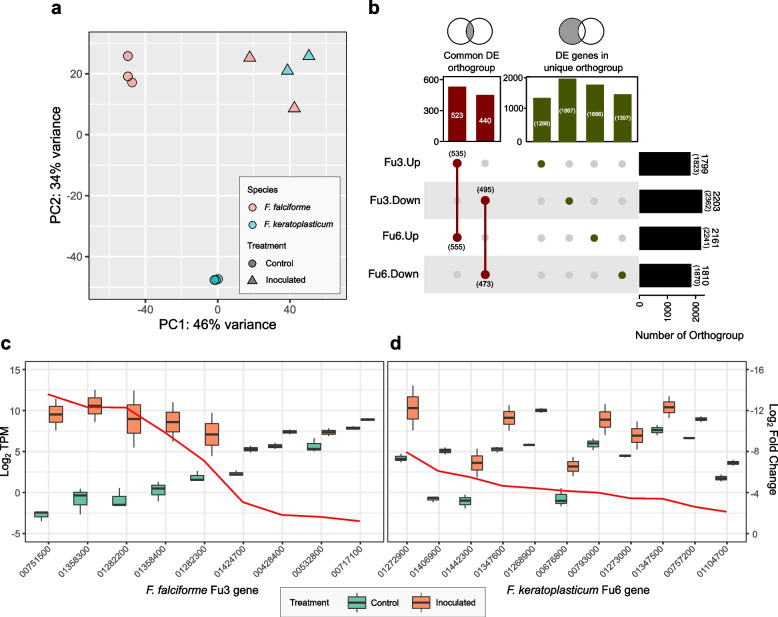
Transcriptomes of *Fusarium solani* species complex inoculated on *Pelodiscus sinensis* eggs. **a** Principal component analysis (PCA) of gene expression patterns in *F. falciforme* Fu3 and *F. keratoplasticum* Fu6 samples. **b** Number of differentially expressed (DE) orthogroup amongst the two pathogens. Numbers in the bracket represents number of genes in the orthogroup. **c**,**d** Expression levels in Log_2_ transcript per million (TPM; left *y*-axis) and Log_2_ fold change (right *y*-axis) of genes containing CFEM domain in *F. falciforme* Fu3 (**c**) and *F. keratoplasticum* Fu6 (**d**) comparing between control (mycelium grown on PDA) and inoculated samples

To better understand how FSSC pathogens infect aquatic animals, we inoculated *F. falciforme* Fu3 and *F. keratoplasticum* Fu6 on *Pelodiscus sinensis* eggs and compared transcriptome-wide gene expression data of both species. The inoculated samples exhibited blotch and membrane phenotypes (Additional file 2: Fig. S18) and were grouped together and distinct from the control samples in the principal component analyses (Additional file 2: Fig. S19 and S20). Globally, both species adopted similar colonisation and infection strategy at a transcriptome level while contacting eggs (Fig. 6a; Additional file 2: Fig. S21; Additional file 3) and we identified a total of 4111 (1823 up- and 2362 downregulated) and 4185 (2241 up- and 1870 downregulated) genes that were differentially expressed (DE; adjusted *p* value < 0.05) in *F. falciforme* Fu3 and *F. keratoplasticum* Fu6 (Fig. 6b), respectively. When considering these genes regardless of expression level, each pathogen underwent differential responses during egg infection—GO enrichment revealed that *F. keratoplasticum* Fu6 exhibited reactions related to pathogenicity such as responses to host, stimulus, and toxic substances, cell adhesion, and regulation of immune system whereas *F. falciforme* Fu3 was mostly ribosome-associated processes such as biogenesis, maturation, and transport (Additional file 1: Table S14). We speculated a larger proportion of genes to be differentially expressed in FCCs and LSCs but observed a reverse pattern where the majority of such genes were located in CCs (Additional file 2: Fig. S22; chi-squared test: Fu3: ${\chi}_1^2$= 10.7, *p* = 0.005; Fu6: ${\chi}_1^2$= 205.84, *p* < 0.001). For instance, only 114 and no genes were DE in LSCs of *F. falciforme* Fu3 and *F. keratoplasticum* Fu6, respectively, indicating genes in FCCs and LSCs were not necessarily enriched during animal pathogenesis.

Several genes that have been previously identified to be involved in *Fusarium*-plant infection were upregulated in the egg-inoculated samples (Additional file 1: Table S15 and S16). The majority of these genes were predicted as effectors or contained a signal peptide. Of particular interest, genes annotated to contain a CFEM (Common in Fungal Extracellular Membrane) domain, a cysteine-rich protein domain found in diverse phytopathogenic fungal species, comprised some of the most DE genes upregulated in both *F. falciforme* Fu3 and *F. keratoplasticum* Fu6 treatments (11 and 15 genes, respectively; Fig. 5c, d). Other examples included the effectors necrosis-inducing proteins (NPP1) and cerato-platanin (CP), which were required for virulence of *F. oxysporum* [46, 47]; ABC membrane and transporter, cytochrome P450 or termed pisatin demethylase (PDA), pectate lyases, whose deletion or inactivation reduced the virulence of *F. vanettenii* FSSC11 on pea [27, 48, 49]. The results suggested that a similar repertoire of genes was utilised during infection regardless of host types by *F. falciforme* and *F. keratoplasticum*.

**Fig. 7:**
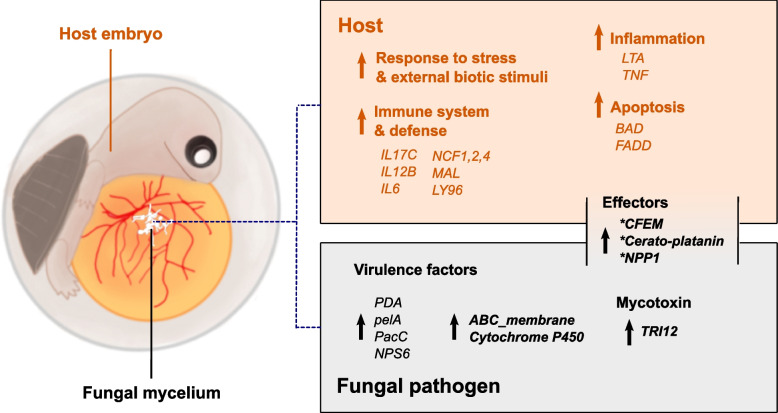
Schematic diagram summarising genes and functions involved in *Fusarium solani* species complex-*Pelodiscus sinensis* egg infection. The colour-coded text represents turtle host (brown) and pathogens (grey). Up arrow denotes upregulated genes and enriched functions. Asterisk denotes the presence of signal peptide

In addition, a total of 535 and 555 genes from *F. falciforme* Fu3 and *F. keratoplasticum* Fu6 treatment, respectively belonging 523 OGs were co-upregulated (Fig. 6b). GO analysis and functional annotations of these genes revealed that both pathogens interacted with the host by positive regulations of their immune system processes and defence responses (Additional file 1: Table S17). These genes include a TRI12 encoding a major facilitator superfamily protein (MFS_1) involved in the export of mycotoxin trichothecene [50], a nonribosomal peptide synthetase (NPS6), a fungal effector involved in secondary metabolite biosynthesis producing AM-toxin, and PacC, a transcription factor-dependent of pH during pathogenesis (Fig. 7; Additional file 1: Table S18 and S19) [51, 52]. In contrast, 440 OGs downregulated in pathogens during egg infection were involved in transmembrane transports of metal ions, spore development, and growth (Additional file 1: Table S20). Furthermore, GO-enriched biological processes of species-specific upregulated genes were similar to GO enrichment of all upregulated DE genes in each pathogen species. Upon examination of these upregulated species-specific genes, it was found that 25.7 and 50.4% of the protein domains in genes of *F. falciforme* Fu3 and *F. keratoplasticum* Fu6 respectively, were present in the co-upregulated DE genes of both species (Additional file 1: Table S21 and S22), suggesting similar infective mechanism were adopted by both pathogen species in animal pathogenicity despite diverged gene sequences.

### Gene expression profile of FSSC-infected animal host

Combined with transcriptome data of various development stages in the *P. sinensis* embryos [53], the presence (75.0–81.5%) of *F. falciforme*- and *F. keratoplasticum*-inoculated turtle RNA-seq reads in samples allowed us to determine host responses to the two pathogens (Additional file 1: Table S23). A large difference can be seen between the gene expression pattern of the host inoculated by FSSC and the natural developing host embryo on similar egg incubation days (PC1: 95% variance; Additional file 2: Fig. S23a), signifying host responded distinctly to FSSC infection. Besides, gene expression in host inoculated with either *F. falciforme* Fu3 and *F. keratoplasticum* Fu6 were highly correlated (*R*^2^ = 0.98, *p* < 0.0001; Additional file 2: Fig. S23b), suggesting the turtle host responded similarly to FSSC pathogens. In addition to genes that were related to embryo development, other upregulated genes were enriched in biological processes involving host immunity, response, and defence to another organism (Additional file 1: Table S24). Specifically, positive regulations of the immune response towards stress and external biotic stimulus were detected. These regulations included leukocyte activation, cytokine production, apoptotic process, and defence response (Fig. 7).

## Discussion

Understanding the genetic diversity within FSSC and their ability to infect cross-kingdoms is of fundamental evolutionary interest and essential for the management of emerging wildlife diseases. Here, we produced highly contiguous assemblies for five FSSC species, established the first *Fusarium*-aquatic animal infection model which utilised high-throughput sequencing technology on Chinese softshell turtle (*Pelodiscus sinensis*) egg, and examined gene expression patterns of *F. falciforme* and *F. keratoplasticum* during infection on the animal host. We uncovered diverse evolutionary dynamics of FSSC chromosomes, and these variations were not associated with egg infection. The availability of these genomes serves as a useful reference for information regarding *Fusarium* evolution and FSSC opportunistic infection on animal host.

Comparative analyses of the six chromosome-level FSSC assemblies allowed the distinction of multiple chromosome compartments based on various structural features. Regions of CCs were highly conserved amongst the FSSC genomes and large synteny were also detected in other non-FSSC species such as *F. oxysporum*, *F. graminearum*, and *F. fujikuroi*, indicating these are the CCs across *Fusarium* genera. When Fokkens et al. [38] compared 59 *F. oxysporum* strains, they coined the term ‘fast-core chromosome’ (FCC), which referred to the three smallest core chromosomes (chr. 11, 12, and 13) that contained features that were intermediate between core and lineage-specific chromosomes. Of these three, two chromosomes shared ancestries with the FCCs identified in our study between FSSCs, suggesting that these chromosomes were already evolving differently compared to CCs since the last common ancestor of *Fusarium* species. The FCCs were gene-rich, enriched in the number of effectors, CAZYmes and SMBGCs, and reduced in repeats compared to LSCs, but had a higher SNP density [38] and were less conserved at strain- and species-level comparisons than CCs. We hereby confirm and verify multi-compartmentalization in FSSC genomes includes fast-core chromosomes in addition to the more commonly discussed ‘two-speed genome’ comprising core and lineage-specific chromosomes in fungal pathogens [25, 26, 54].

Although the repeat portion of the fungal genome is typically accompanied by a higher abundance of methylated DNA bases [43], we found methylation was uniformly reduced along repetitive content of LSC of FSSC genomes. Enriched H3K27me3 and an increased methylation level were independently found in the FCCs of *F. oxysporum* [38] and FSSC, respectively. In *Arabidopsis thaliana*, H3K27me3 was exclusively associated with DNA methylation at transposable elements [55]. We speculate enrichment of both marks may be the hallmarks of all *Fusarium* FCCs, and the interplay of DNA methylation and H3K27me3 may mediate transcriptional responses in the enriched gene families involved in the pathogenicity processes [56]. Chromosomes harbouring similar features have been reported in other *Fusarium* species [38, 54], and additional sequencing may identify and elucidate the molecular mechanisms of FCCs as they displayed differential evolutionary dynamics in each species. DNA methylation levels of FSSC genomes were surprisingly higher than most previously determined ascomycetes in Bewick et al. [43], which coincides with the only exception of high methylation, *Pseudogymnoascus destructans*, which is also an animal pathogen. Further comparisons are needed to elucidate the potential association between DNA methylation and animal virulence in fungal pathogens.

Some FSSC species are model systems for cell biology [57], biocatalytic applications [58], and the most extensively studied plant pathosystem that involves *F. vanettenii* on pea [59]. Our attraction assays indicated no signs of egg attraction suggesting chance-encounter of FSSC pathogens on eggs, unlike some pathogens that seek hosts actively in the environment [60, 61]. During plant infection, hyphae of fungal pathogens penetrate host tissue through natural openings such as stomata or lenticels [62]. We observed the initial stage of disease development which include spore germination, hyphal extension, and colonisation of *F. falciforme* and *F. keratoplasticum* on turtle eggs, both on the eggshell and internal embryo. While eggshell serves as a protection for the developing embryos, we observed both FSSC pathogens were capable of invading egg inclusions through the eggshell and caused tissue degradation of egg membrane. Previous study had also shown calcium depletion of sea turtle eggshell post-infection by *F. solani* [63], suggesting lytic activity of FSSC pathogens. Nevertheless, the eggshell of *P. sinensis* is thicker than sea turtle eggshell with an expanded calcareous layer [64], emphasising that egg penetration may have played a more primary role in establishing infection at least for the *P. sinensis* host.

Conserved pathogenicity traits such as effectors in pathogen of different strains might play a role in infecting the same host [65]. In our egg infection experiment, only a few differences were found between gene expression and overall regulated biological processes of both FSSC pathogens. Interestingly, we detected that plant pathogenicity-related genes were also upregulated in animal infection. These include carbohydrate-active enzymes such as cellulase which functions to degrade plant cell wall materials, and pathogenicity genes containing CFEM domain, which were few of the top most expressed genes in both pathogens. CFEM is found in diverse phytopathogenic fungal species with various virulent roles such as appressoria formation in *Pyricularia oryzae* (syn. *Magnaporthe oryzae*) [66] and root colonisation in *F. oxysporum* [67]. The most possible explanation for its role in animal infection is biofilm formation, as seen in *Candida albicans* [68] and white blotches surrounding the embryo. Nevertheless, the role of CFEM domain and plant virulence genes in animal infection remained to be elucidated. This combined evidence suggested that FSSC species may adopt a similar repertoire of genes in establishing infection across kingdoms. Intriguingly, genes involved in pathogenesis are usually enriched and highly expressed during the infection process in the FCCs or LSCs of *Fusarium* species [11, 38, 42, 69, 70] but we found no such association in *F. falciforme* and *F. keratoplasticum* during the egg infection. The differences suggest that the evolution of FSSC genomes may be shaped by lifestyles other than animal parasitism and that the differentially expressed genes during the infection process thus represented a more generic infection process.

## Conclusions

In this study, we identified three compartments in the genomes of the *Fusarium solani* species complex (FSSC) that appear to be the hallmarks of *Fusarium* chromosome evolution. Moreover, the reference quality FSSC genomes offer prospects for future research in pathogenesis regardless of the host species. We present a landscape of transcriptome profiling of FSSC genes during egg infection, some of which were also highly expressed when infecting plant hosts. The combined results provided new insights into the genomic characteristics of animal-infecting FSSC species and their disease development, particularly on turtle eggs. This research represents the beginning of critical steps to understanding FSSC infection on turtles towards data-informed decision making and management of disease epidemics to reduce disease occurrences in the wild and man-managed settings.

## Methods

### Nucleic acid isolation, genome and transcriptome sequencing

Six isolates from FSSC clade 3 were chosen for genome sequencing and comparative analyses (see Additional file 3 for fungal culturing conditions and species identification of isolates) [14, 22, 71]. Fresh mycelium from the cultures was scraped off from the culture media for gDNA and RNA isolation. Genomic DNA was extracted following protocols designed for high-molecular-weight gDNA sequencing [72] and size selection and purification of isolated gDNA was performed following [73]. The integrity of gDNA was evaluated using Fragment Analyzer 5200 (Advanced Analytic Technologies, Inc., Ankeny, USA) and the fragment lengths were determined using PROsize 2 software (Advanced Analytic Technologies, Inc., Ankeny, USA). Genomes were sequenced using both Illumina and Oxford Nanopore platforms. Detailed information such as sequencing platforms, library kits used, and sequence accession number for each sample can be found in Additional file 1: Table S25. The summary of DNA sequencing data is shown in Additional file 1: Table S1. For gene model prediction and annotation, RNA of the six isolates were extracted following the TRIzol reagent protocol (Thermo Fisher Scientific, Waltham, MA, USA, Cat. #15596026). The integrity of the isolated RNA was checked using 1.5% agarose gel, and quantity was determined using Invitrogen Qubit® 4 Fluorometer (Thermo Fisher Scientific, Waltham, MA, USA) before library preparation and sequencing (Additional file 1: Table S25).

### Genome assembly and annotations

Guppy (v3.2.4 and v4.0.11; Oxford Nanopore Technology) or albacore (for 1D^2^ reads; v2.2.7; Oxford Nanopore Technology) were used to perform basecalling of Nanopore raw signals (Additional file 1: Table S1) and assembled using Flye (v2.5) [30] and further polish and correct the assembled sequences with consensus mapped reads from Illumina using Pilon (v1.22) [74]. Haplotypes were collapsed with HaploMerger2 (ver. 20180603) [75]. Genome haploid length was estimated via GenomeScope 2.0 [76]. Annotation of repeat elements of the genomes was performed following Berriman et al. [77] except described otherwise in the following: a consensus repeat library was created using repeat elements identified via RepeatModeler (v.open-1.0.8) and TransposonPSI (release 08222010; http://transposonpsi.sourceforge.net/) and merged using usearch (v8.1.1861) [78]. RepeatMasker (v.open-4.0.7; option -s; https://www.repeatmasker.org) was used to mask the predicted repeat regions in each genome. Telomeric repeats of each scaffold were determined using Tandem Repeat Finder (v4.09; default parameters) [79]. Enriched hexamers were identified (TTAGGG) manually using Python script on the terminal 5 kb regions of each scaffold to confirm the presence of telomeres. Genes of the assemblies were predicted using AUGUSTUS (v3.3.3) [80] and trained with BRAKER2 (option fungi and softmasked; v2.1.4) [81]. MAKER2 (v2.31.9) [32] was then used to combine evidence from the assembled transcripts, reference proteomes, and transcript evidence obtained from RNA-seq of the mycelium to produce a final gene annotation set. Completeness of each genome was accessed by BUSCO (v5.2.2; Table 1) [33]. Functional annotations of the amino acid sequences were carried out using eggNOG-mapper v2 (http://eggnog-mapper.embl.de; default parameters) [34] on eggNOG v5 database [82]. Protein domains and families were identified using pfam_scan.pl (v1.6; http://ftp.ebi.ac.uk/pub/databases/Pfam/Tools/) on Pfam database (release 34.0) [83]. Additional annotations were carried out as follows: carbohydrate-active enzymes were determined using dbCAN2 (v2.0.11) [84]; secondary metabolite detection via antiSMASH (v6.0) [85]; and fungal effectors were predicted using EffectorP (v3.0) [86] on amino acid sequences which passed the signal peptide prediction via signalP (v6.0) [87].

### Comparative genomic analyses

Orthology was assigned using OrthoFinder (v2.3.8) [35] with proteomes of six species from this study, 17 other *Fusarium* published assemblies from and an outgroup species *Beauveria bassiana* (Additional file 1: Table S2). A total of 2385 single-copy orthogroups (OG) were determined from proteomes of 24 species and were aligned using MAFFT (v7.487; option -maxiterate 1000) [88]. The alignments from each of the OG were sent to RAxML-NG for maximum likelihood phylogeny inference (v0.9.0; option --model LG+I+F+G4, --bs-trees 100) [89]. The maximum likelihood trees and bootstrap-supported replicates generated from the previous step were combined for the construction of consensus species tree using ASTRAL-III (v5.7.7; option -r 100) [90]. Lastly, a maximum likelihood phylogeny from concatenated amino acid alignments of single-copy OG was built using RAxML-NG (v0.9.0; option --model LG+I+F+G4, --bs-trees 100) [89] with 100 bootstrap replicates. For *d*_N_/*d*_S_ analysis, codon alignments of one-to-one orthologs between *F. falciforme* Fu3 and *F. keratoplasticum* Fu6 were produced using TranslatorX (v1.1) [91]. Ratio of *d*_N_/*d*_S_ were calculated for each ortholog alignment using PAML^2^ of CODEML programme (v4.9e; option runmode=-2, seqtype=1, CondonFreq=3, and fix_omega=0) [92].

### Core, fast-core, and lineage-specific chromosomes assignment

Synteny analysis between FSSC species (i.e. *F. falciforme* Fu3 vs. *F. vanettenii* 77-13-4) and between FSSC and non-FSSC species were carried out using MUMmer4 [93] and PROmer (v.3.23) [94], respectively (see Additional file 3). From the synteny analysis, a total of 12 conserved linkage groups were assigned unambiguously in the six FSSC genomes, and these were defined as core chromosomes. Lineage-specific chromosomes were assigned based on lack of synteny regions linked via one-to-one gene with Fu3 chromosomes (Additional file 2: Fig. S5). Contigs of less than 450kb were not included in this assignment due to an unclassifiable region via single-copy orthologues or genome alignment via nucmer [93]. The excluded contigs comprised 0.3–3.5% of assemblies and ranged from 11 to 404 genes. The designated lineage-specific chromosome includes chromosomes 13 and 14 of Fu3, chromosomes 13, 14, and 15 of Fu6 and Ph1, chromosomes 13, 14, 15, and 16 of LHS11, chromosomes 13 to 19 of LHS14, and chromosomes 13, 14 to 23 of Fs6. From the core chromosomes, fast-core chromosomes were designated based on higher proportion of FSSC-specific gene (Fig. 2d). FSSC-specific gene were defined as genes with no orthologs identified in *F. fujikuroi* IMI58289, *F. graminearum* PH-1, and *F. oxysporum* f. sp. *lycopercisi* 4287 genomes [24, 40, 95]. The designated fast-core chromosomes include chromosomes 7, 11, and 12 of all six FSSC assemblies.

### Methylation analysis

To examine 5-methylcytosine (5mC) in DNA sequences, Nanopore raw FAST5 files of *F. falciforme* Fu3, *F. keratoplasticum* LHS11, *Fusarium* sp. Ph1, and *F. vanettenii* Fs6 were used to run Megalodon (v2.3.3; Oxford Nanopore Technologies) and Guppy (v5.0.11; Oxford Nanopore Technologies) using the default parameters. To determine the methylation level of each CpG site, we calculated the ratio of methylated reads including both strands. Calculation of methylation levels was carried out using a 10-kb window.

### Animal inoculation experiments

We incubated softshelled turtle (*Pelodiscus sinensis*) eggs up to day 30, at which point the embryo was estimated to have developed to either stage TK21 to 22 [96]. Dead eggs were removed, and alive eggs were kept for experiments which included pathogen inoculation, host attraction assay, and eggshell observations. To determine if the pathogens *F. falciforme* Fu3 and *F. keratoplasticum* Fu6 can be attracted to the egg host, an attraction assay experiment was performed and hyphal growth towards the host was measured. Eggshell fragments inoculated with either *F. falciforme* Fu3 or *F. keratoplasticum* Fu6 pathogens were collected for histological observations using scanning electron microscopy (SEM) and laser scanning confocal microscopy (LSCM) for invasion route. For transcriptome sequencing of FSSC infection on *P. sinensis* eggs, we injected 10^7^ spores/mL suspension into the eggs and incubated it for another 3 to 4 days before RNA isolation. Detailed methods can be found in Additional file 3.

### Total RNA isolation, library preparation, and sequencing

RNA isolation of the animal inoculation experiment was carried out according to the TRIzol reagent protocol. At the cell lysis step, each sample in 2mL screw-cap tube was mixed with six to eight 0.8-mm stainless steel beads, flash-freezing the tubes in liquid nitrogen, and homogenised using PowerLyzer24 homogeniser (MoBio Laboratories, Carlsbad, USA, Cat. #13155) set to 3000×*g* for 20 s and repeated at least twice to ensure the sample was homogenised. Isolated RNA was quantified using Invitrogen Qubit® 4 Fluorometer (Thermo Fisher Scientific, Waltham, USA) and checked for integrity in 1.5% agarose gel. Samples with adequate concentration and show no degradation was chosen for RNA sequencing. In total, 20 samples were sequenced, which include three *F. falciforme* Fu3 and three *F. keratoplasticum* Fu6 positive controls (mycelium from PDA media), seven *F. falciforme* Fu3 and seven *F. keratoplasticum* Fu6 inoculated samples (Additional file 1: Table S26). The inoculated samples were made up of four blotch and three membrane samples. Paired-end library was prepared for the RNA samples using NED NextⓇ Ultra™ RNA Library Prep Kit and sequenced on Illumina NovaSeq 6000 instrument with 150 bp paired-end reads.

### Analysis of RNA-seq reads

Raw RNA reads were trimmed to remove adaptor sequences and poor-quality reads using fastp (option -l 30; v0.20.1) [97]. Trimmed reads of each sample were mapped to their respective genome (*F. falciforme* Fu3 or *F. keratoplasticum* Fu6) according to the inoculation treatment and the host *P. sinensis* genome (GCF_000230535.1_PelSin_1.0) [53] using STAR (v2.7.7a) [98]. To ensure RNA reads which mapped on the *Fusarium* genomes were not from the host, reads mapped onto both *Fusarium* and host genome and had lower CIGAR scores in *Fusarium* were excluded from further analyses. Raw read count of each gene was calculated using featureCounts (-p -s 2; v2.0.1) [99]. We first examined the inoculated samples from the blotch and membrane to see if they had a dissimilar gene expression pattern through principal component analysis (PCA) and found that 43% of the variances could be explained between these two sample types in either treatment (Additional file 2: Fig. S19). Since our aim is to determine the general pattern of FSSC and animal pathogenesis, we grouped the blotch and membrane samples as ‘inoculated samples’ for further analyses. Differentially expressed genes (DEGs) between inoculated and control samples in *F. falciforme* Fu3 and *F. keratoplasticum* Fu6 were inferred using DESeq2 (*padj* < 0.05; v1.24) [100]. Functional enrichment of the DEGs was identified using the ‘topGO’ (v2.36.0) [101] package. We used the same pipeline as described for the analysis of the host’s RNA reads to determine the DEGs of the inoculated host using the *P. sinensis* genome [53] as reference. For comparison, the RNA-seq dataset of *P. sinensis* embryos from different embryo developmental stages (TK19 and TK23 defined as in [96] generated by [53]) was chosen as the control because our samples were collected between TK21 to 22. Of those, 12,419 genes were differentially expressed (adjusted *p* value < 0.05), with 5815 up- and 6604 downregulated genes during the infection experiment.

### Statistical analyses

We used R package ‘ggpubr’ [102] to perform Wilcoxon rank sum test for mean comparisons of groups in analyses which include d*N*/d*S*, proportions of repeat content and FSSC-specific genes, methylation levels, and host attraction assay. For correlation analyses, we used R package ‘stats’ [103] Kendall’s Tau method to compare methylation levels amongst the genome features and used Pearson method to compare orthologous gene expression between *F. falciforme* Fu3 and *F. keratoplasticum* Fu6. Chi-squared test of R package ‘stats’ [103] was used to compare proportion of differentially expressed genes in different chromosome types.

## Supplementary Information

**Additional file 1: Table S1.** Read summary of genome assemblies from each sequencing platform. **Table S2.** *Fusarium* genomes and an outrgroup genome used in the phylogenomic analysis and comparative studies. **Table S3.** Scaffold assembly completeness. **Table S4.** FSSC genome assemblies used to compare N90. **Table S5.** Number of genes with predicted signal peptide and effector using SignalP and EffectorP, respectively. **Table S6.** Number of genes with predicted carbohydrate-active enzymes (CAZymes) and its categories using dbCAN2. **Table S7.** Number of secondary metabolites biosynthetic gene cluster detected via antiSMASH. **Table S8.** Type and presence of secondary metabolite products in each FSSC genomes predicted via antiSMASH. **Table S9.** *Fusarium solani* species complex (FSSC) sequences used in multi-locus sequence typing (MLST) phylogenetic tree in Fig. S2. **Table S10.** Structural features of FSSC genome assemblies. **Table S11.** Gene number and proportion of effector, carbohydrate active enzymes and secondary metabolite gene cluster in each FSSC chromosome. **Table S12.** Average number of genes predicted to encode for effector, carbohydrate-active enzymes (CAZYmes) and secondary metabolite biosynthetic gene clusters (SMBGCs) of FSSC genomes by chromosome types. **Table S13.** Multiple mean comparisons of methylation levels among the chromosome type and genome features in *F. falciforme Fu3*. **Table S14.** Top 50 enriched biological processes of FSSC pathogen *F. falciforme* Fu3 and *F. keratoplasticum* Fu6 during *P. sinensis* host infection. **Table S15.** Subset of differentially expressed and upregulated genes of *F. falciforme* Fu3 during *P. sinensis* host infection previously known to associate with plant host infection. **Table S16.** Subset of differentially expressed and upregulated genes of *F. keratoplasticum* Fu6 during *P. sinensis* host infection previously known to associate with plant host infection. **Table S17.** Selected enriched biological processes of co-upregulated genes of *F. falciforme* Fu3 and *F. keratoplasticum* Fu6 during *P. sinensis* host infection. **Table S18.** Top 50 differentially expressed genes of *F. falciforme* Fu3 which were co-upregulated with *F. keratoplasticum* Fu6 (Table S19) during *P. sinensis* host infection. **Table S19.** Top 50 differentially expressed genes of *F. keratoplasticum* Fu6 which were co-upregulated with *F. falciforme* Fu3 (Table S18) during *P. sinensis* host infection. **Table S20.** Selected enriched biological processes of co-downregulated genes of *F. falciforme* Fu3 and *F. keratoplasticum* Fu6 during *P. sinensis* host infection. **Table S21.** Top 50 differentially expressed genes of *F. falciforme* Fu3 which were unique to *F. keratoplasticum* Fu6 (by orthogroup) during *P. sinensis* host infection. **Table S22.** Top 50 differentially expressed genes of *F. keratoplasticum* Fu6 which were unique to *F. falciforme* Fu3 (by orthogroup) during *P. sinensis* host infection. **Table S23.** RNA-seq and read statistics of FSSC-animal infection experiment. **Table S24.** Upregulated biological processes of *P. sinensis* host infected by pathogen *F. falciforme* and *F. keratoplasticum*. **Table S25.** Sequencing information of gDNA and RNA for genome assembly and gene annotation. **Table S26.** All sequenced samples and metadata for inoculation experiment.

**Additional file 2: Fig. S1.** Genome features in FSSC assemblies. **Fig. S2.** Multi-locus sequence typing (MLST) phylogeny tree of FSSC. **Fig. S3.** Genome phylogeny of *Fusarium* species. **Fig. S4.** Orthologue sharing amongst *Fusarium* chromosome. **Fig. S5.** FSSC genome synteny. **Fig. S6.** Synteny between *F. vanettenii* Fs6 and FVANE. **Fig. S7.** Location of FSSC-specific genes across genomes. **Fig. S8.** Selection level in FSSC chromosomes. **Fig. S9.** Orthogroup number comparison between *F. falciforme* Fu3 and *F. oxysporum* f. sp. *lycopercisi* 4287. **Fig. S10.** Syntenic dotplot between FSSC and non-FSSC species. **Fig. S11.** Methylation level of each chromosome in FSSC. **Fig. S12.** Methylation analysis of *F. keratoplasticum* LHS11. **Fig. S13.** Methylation analysis of *Fusarium* sp. Ph1. **Fig. S14.** Methylation analysis of *F. vanettenii* Fs6. **Fig. S15.** Gene density in 10 kb window per chromosome or chromosome types. **Fig. S16.** Host attraction assay. **Fig. S17.** Scanning electron microscopy images. **Fig. S18.** Chinese soft-shelled turtle *P. sinensis* egg inoculated with conidia of *F. falciforme* Fu3 or *F. keratoplasticum* Fu6. **Fig. S19.** Principal component analyses of pathogens’ gene expression pattern of inoculated samples. **Fig. S20.** Principal component analyses of pathogens’ gene expression pattern of all samples. **Fig. S21.** Correlation plots of log_2_ normalized transcript per million (TPM) of one-to-one orthologous gene of *F. falciforme* Fu3 and *F. keratoplasticum* Fu6. **Fig. S22.** Distribution of all differentially expressed genes of FSSC pathogens during egg inoculation experiment. **Fig. S23.** Gene expression pattern of the animal host *P. sinensis*.

**Additional file 3.** Extended Results and Methods.

## Data Availability

All sequences generated from this study were deposited on NCBI under BioProject PRJNA782245 [104] and accession number of gene sequences from MLST of isolates can be found in Additional file 1: Table S9.
