# Supplementary material for: Comparative genomic and transcriptomic analyses of trans-kingdom pathogen Fusarium solani species complex reveal degrees of compartmentalization
Source: BMC Biol. 2022 Oct 20;20:236. doi: 10.1186/s12915-022-01436-7 (PMC9583462; doi:10.1186/s12915-022-01436-7)

Additional File 2:
Supplementary Figures

for

Comparative genomic and transcriptomic analyses of trans-kingdom pathogen *Fusarium solani* species complex reveal degrees of compartmentalization

Daphne Z. Hoh[1,2,3], Hsin-Han Lee[1], Naohisa Wada[1], Wei-An Liu[1], Min R. Lu[1], Cheng-Kuo Lai[1,4], Huei-Mien Ke[1], Pei-Feng Sun[1,2,3], Sen-Lin Tang[1,2], Wen-Hsin Chung[5], Ying-Lien Chen[6], Chia-Lin Chung[6] and Isheng Jason Tsai[*1,2,4]

*Corresponding author: Isheng Jason Tsai ijtsai@sinica.edu.tw

This file contains:
Fig. S1 – S23

Fig. S1. Genome features in FSSC assemblies. Proportions of (a) genome features and (b) repeat contents. Number in (b) represent proportion of each repeat classes. Fu3, LHS14 and Fs6 contains 0.1% SINE and is not noticeable in the plot.

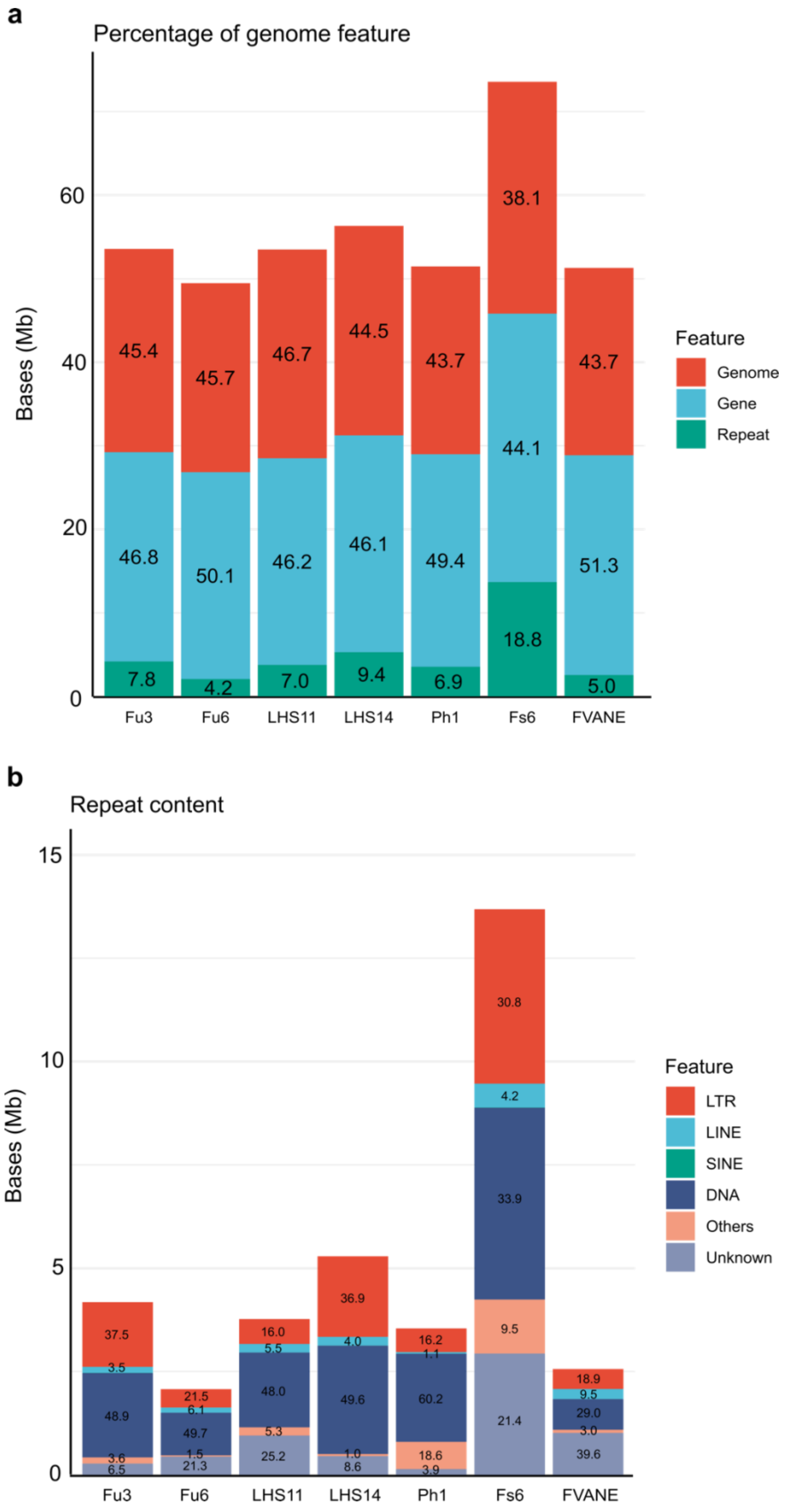

Fig. S2. Multi-locus sequence typing (MLST) phylogeny tree of FSSC. This tree is constructed using ITS, RPB2, and TEF1 regions. Asterisk indicates > 80% bootstraps value. Species name in bold indicates isolate sequenced in current study.

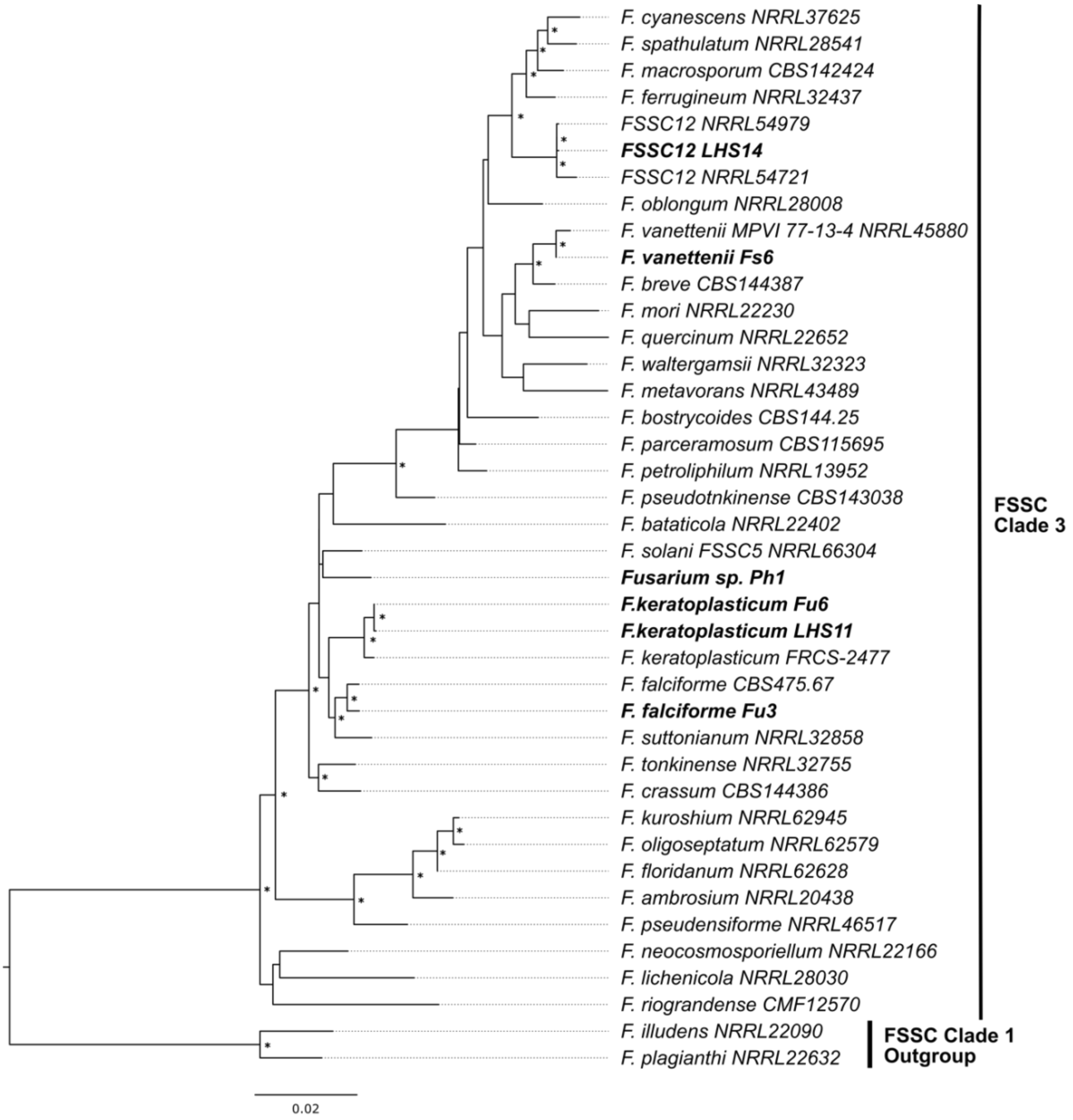

Fig. S3. Genome phylogeny of *Fusarium* species. Phylogeny was constructed using 2,385 single-copy orthogroup sequences in 23 *Fusarium* assemblies and *Beauveria bassiana* as the outgroup. Asterisk indicates 100% bootstrap value. Coalescent unit on scale bar applies on internal branch only. Yellow box denotes the FSSC clade. Species name in bold represents genomes sequenced in current study. Source origin (host) of isolates represented by icons.

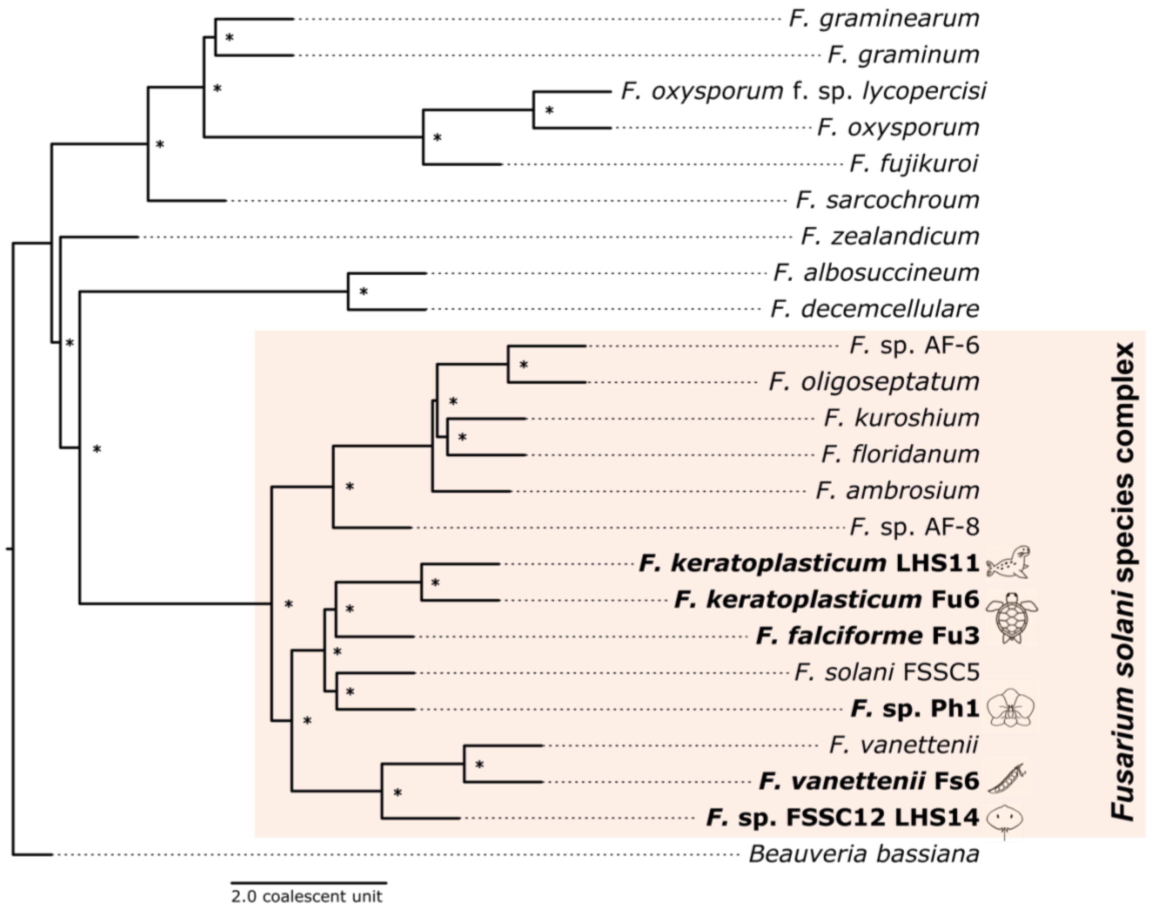

Fig. S4. Orthologue sharing amongst *Fusarium* chromosome. (a) Proportion of one-to-one orthologue shared across each FSSC chromosome. (b) Proportion of one-to-one orthologue with three additional *Fusarium* genomes outside of FSSC including *F. oxysporum*, *F. graminearum*, and *F. fujikuroi*. Red text and line are the reference for proportion level. Asterisk indicates telomere-to-telomere gapless chromosome.

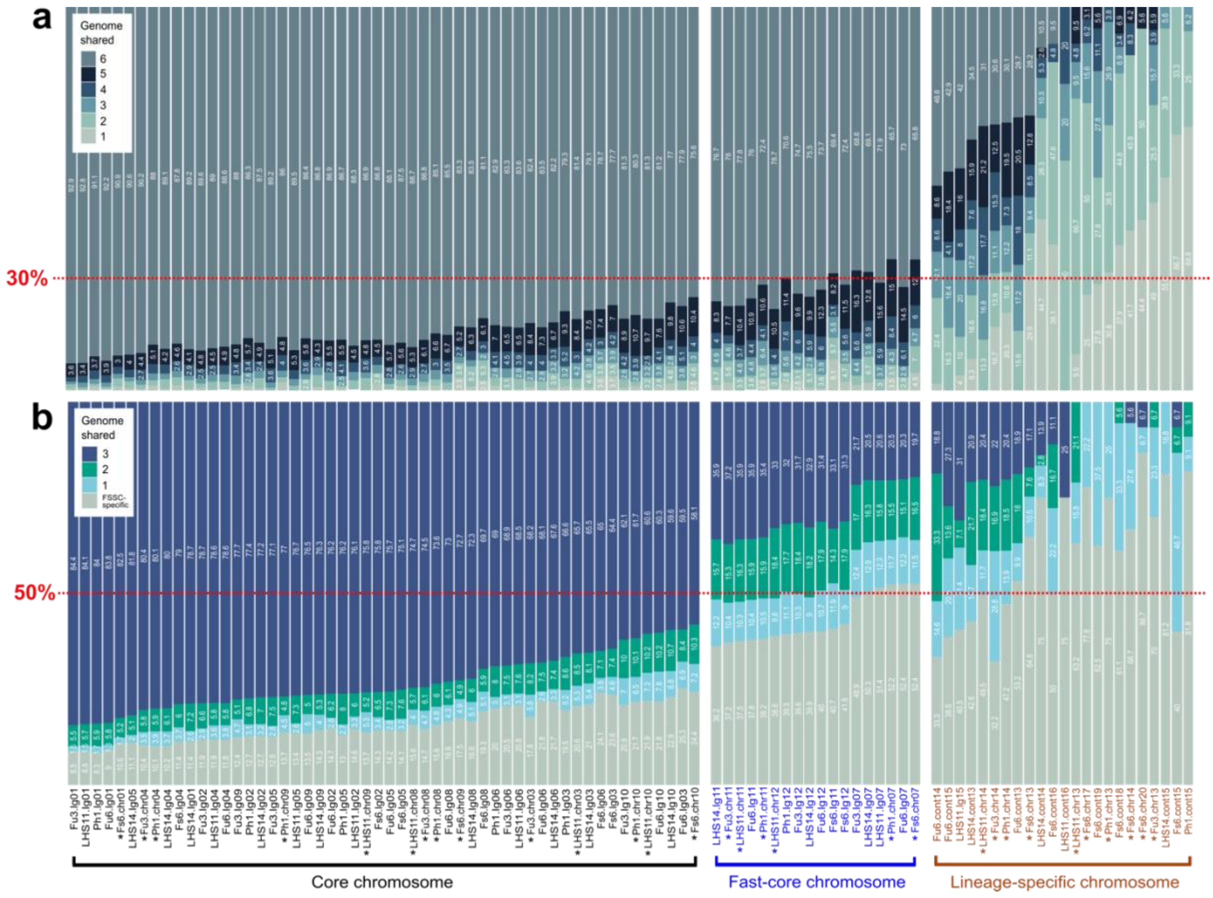

Fig. S5. FSSC genome synteny. Synteny analysis was conducted using single-copy orthologue with *F. falciforme* Fu3 as reference. Colours refer to gene corresponds to Fu3 genome. Single line indicates one gene. Asterisk and dot indicate telomere-to-telomere gapless chromosome and fast-core chromosome, respectively.

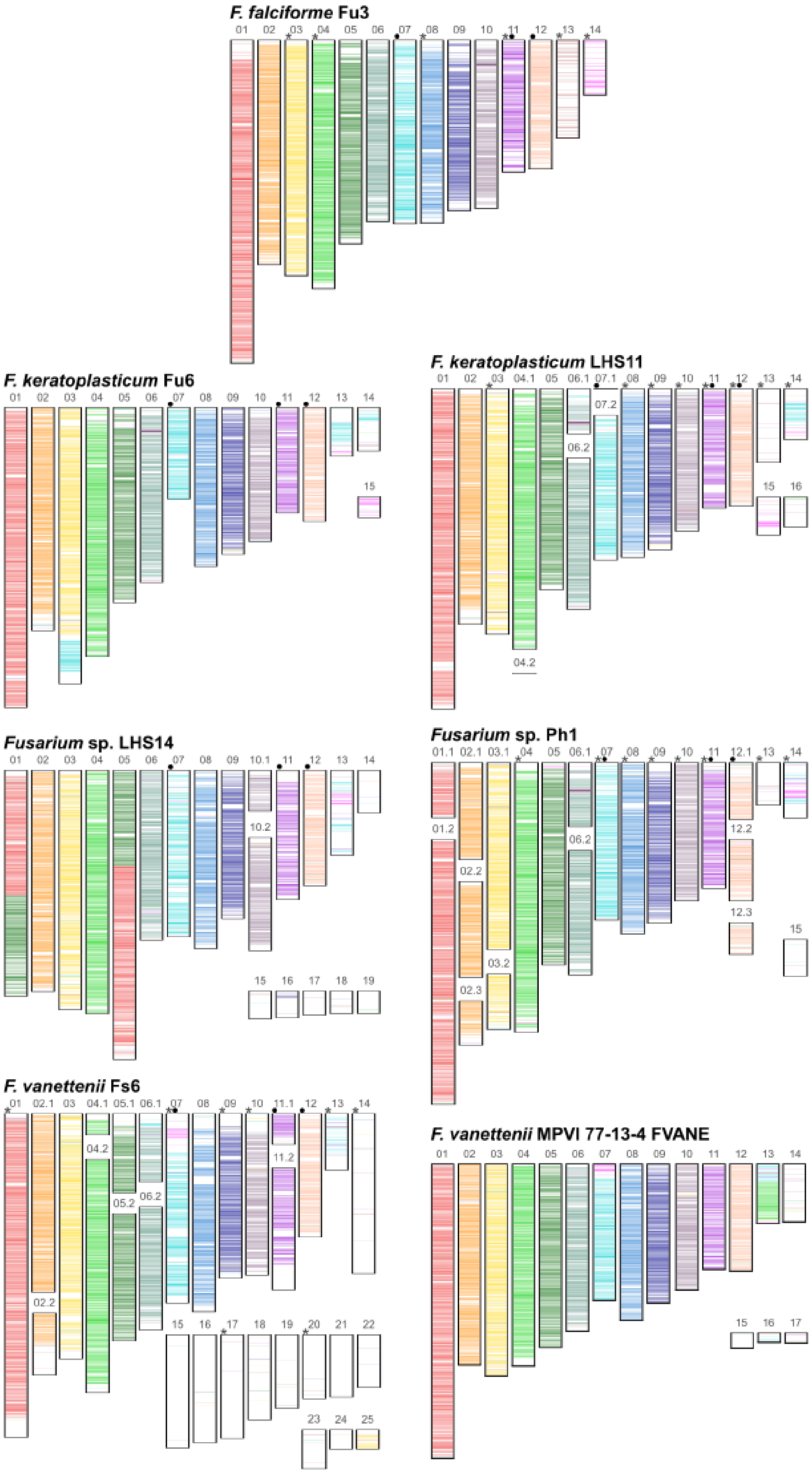

Fig. S6. Synteny between *F. vanettenii* Fs6 and FVANE. In *F. vanettenii* Fs6, the chromosome label in colour black, blue and brown represent core, fast-core and lineage-specific chromosomes, respectively. Asterisk indicate telomere-to-telomere gapless chromosome.

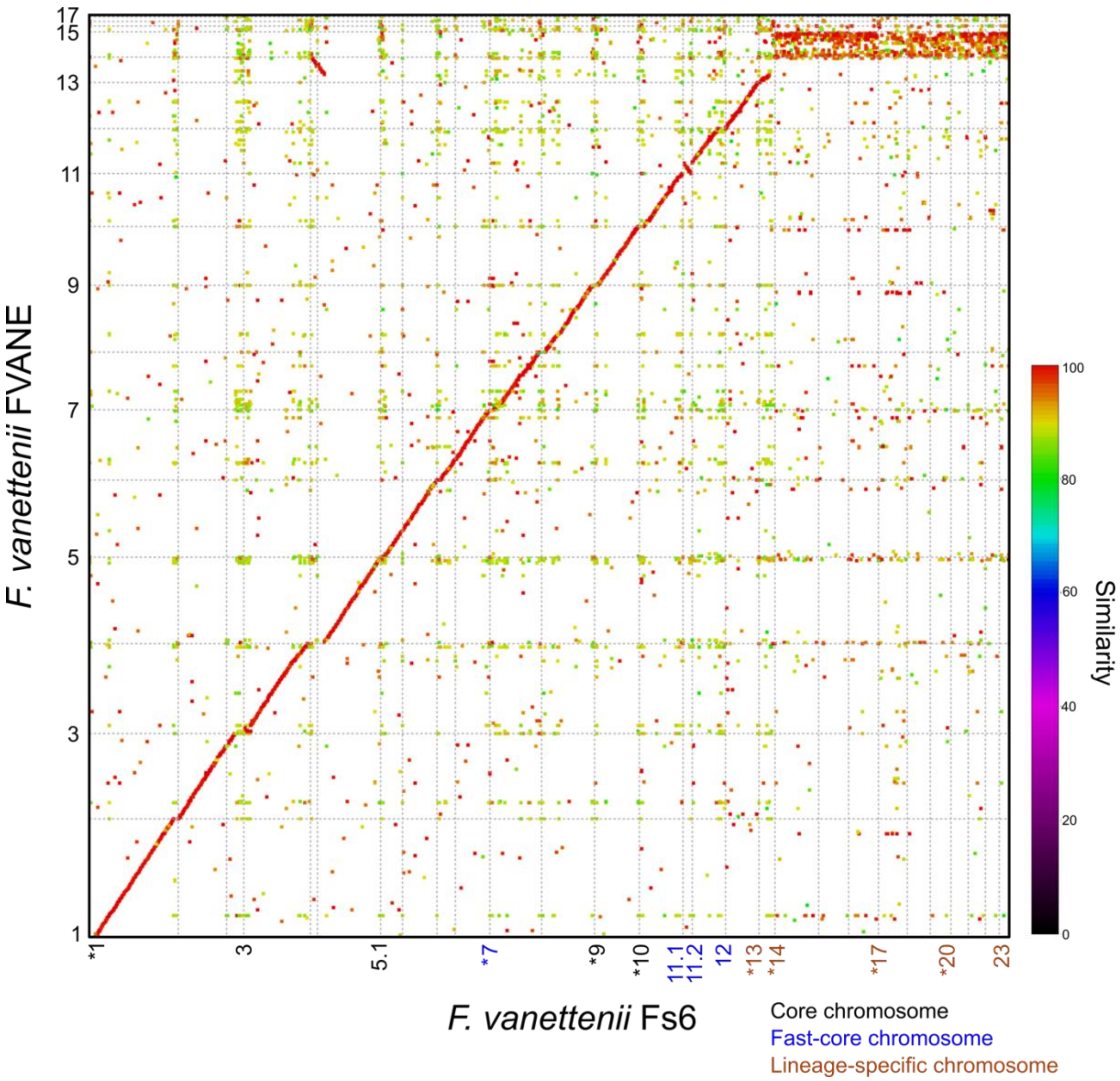

Fig. S7. Location of FSSC-specific genes across genomes. Single red line indicates one gene. Asterisk and dot indicate telomere-to-telomere gapless chromosome and fast-core chromosome, respectively.

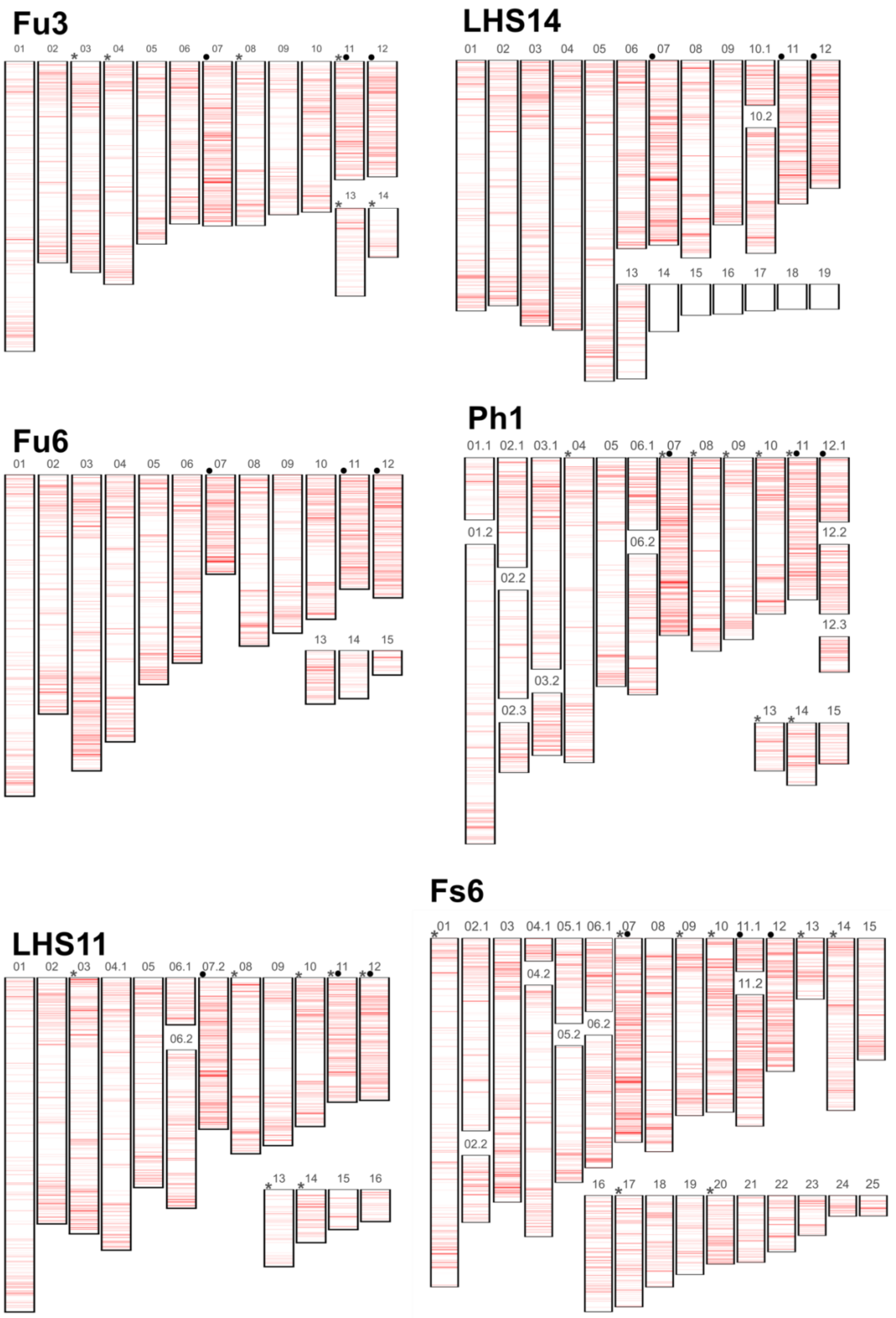

Fig. S8. Selection level in FSSC chromosomes. Estimates of non-synonymous substitutions (d*N*), synonymous substitutions (d*S*) per gene, and their ratio (d*N*/d*S*) comparing each group differentiating location of single-copy orthogroup between *F. falciforme* Fu3 and *F. keratoplasticum* Fu6. Groups compared include gene located on the same corresponding core chromosome 'CC_same' and fast-core chromosome 'FCC_same' while 'Rearranged' indicates genes not located on the corresponding chromosomes. Statistical significance was calculated using Wilcoxon rank-sum test (****: $p < 0.0001$; ns: $p > 0.05$).

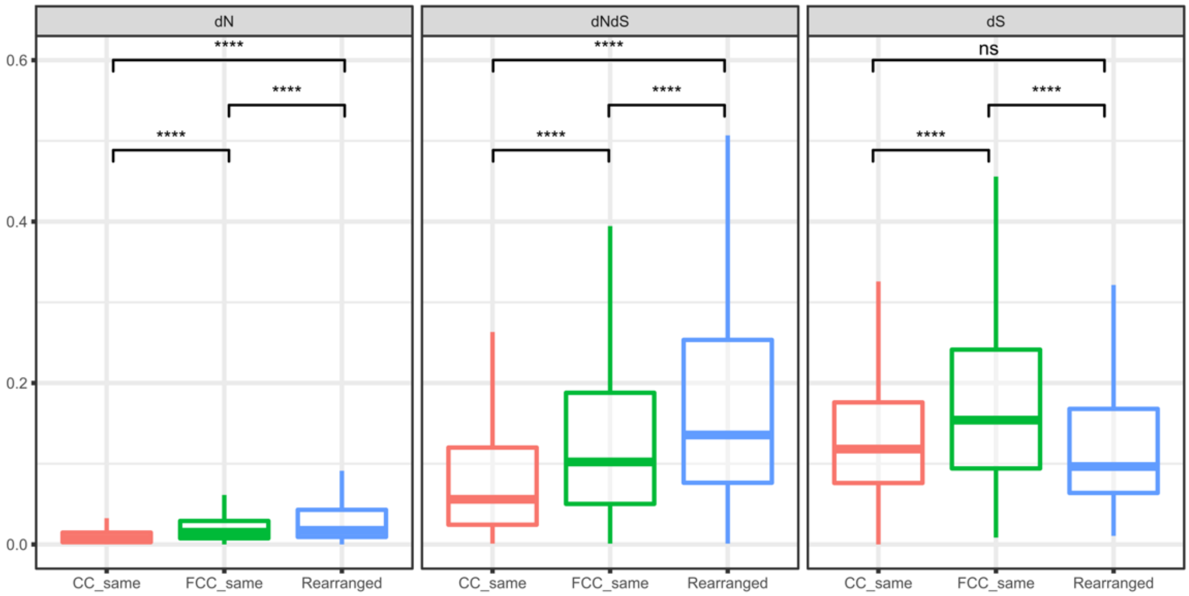

Fig. S9. Orthogroup number comparison between *F. falciforme* Fu3 and *F. oxysporum* f. sp. *lycopercisi* 4287. Pairwise chromosome comparisons of shared orthogroup number (heatmap) and total number of orthologous gene per chromosome (bar plots).

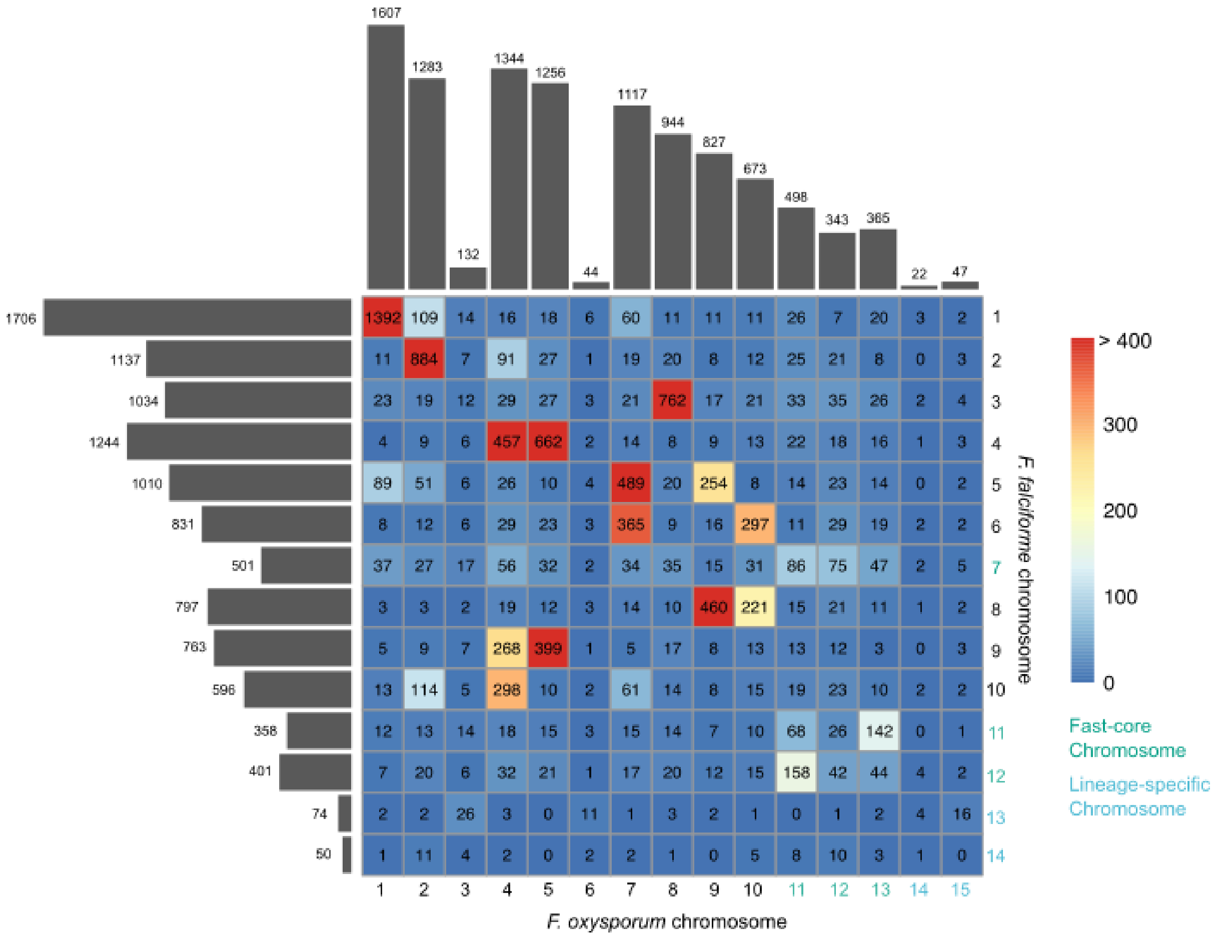

Fig. S10. Syntenic dotplot between FSSC and non-FSSC species. Synteny detected via PROmer comparing between *F. falciforme* Fu3 and (a) *F. graminearum* and (b) *F. fujikuroi*.

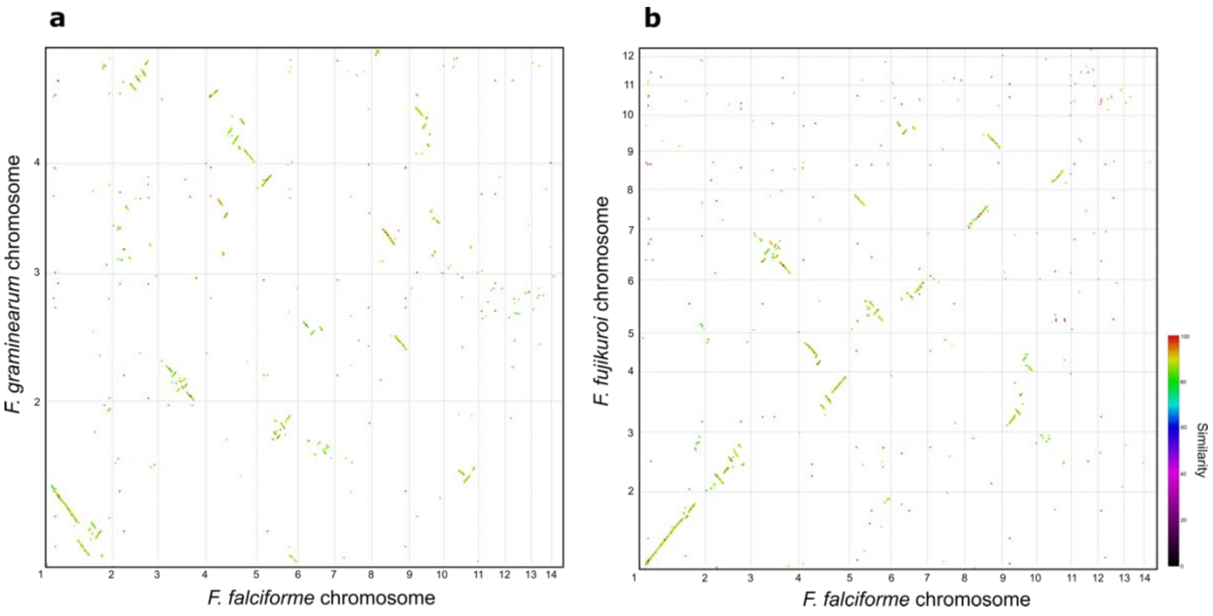

Fig. S11. Methylation level of each chromosome in FSSC. Boxplots from top to bottom: *F. falciforme* Fu3, *F. keratoplasticum* LHS11, *Fusarium* sp. Ph1, and *F. vanettenii* Fs6 genomes.

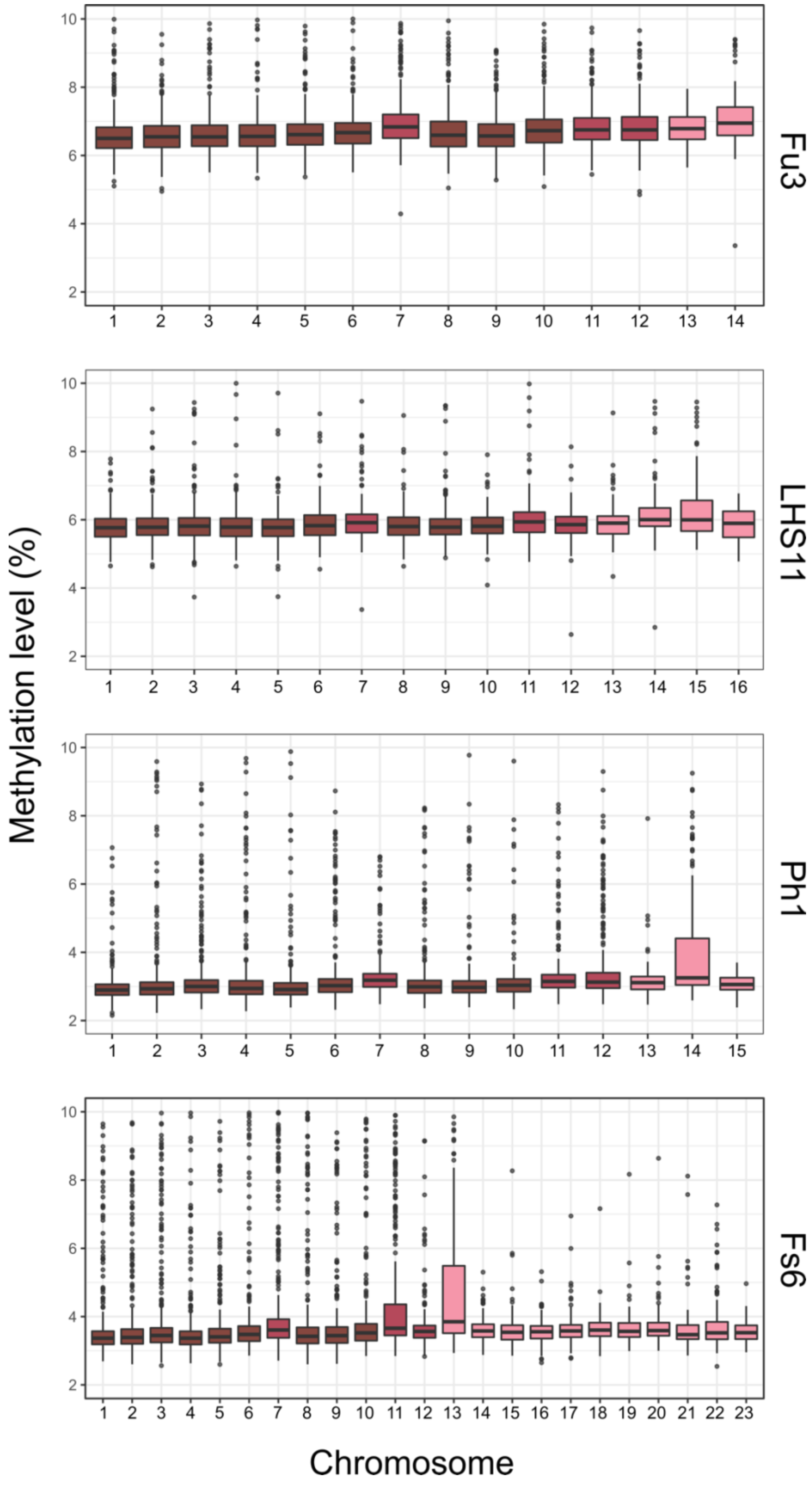

Fig. S12. Methylation analysis of *F. keratoplasticum* LHS11. (a) Percentages of coding gene, repeats, and methylation across 10 kb window of chromosomes. (b) Pearson correlation coefficient between proportion of genome features and methylation. (c) Methylation level of genome features. CC, FCC, and LSC represents core chromosome, fast-core chromosome, and lineage-specific chromosome, respectively.

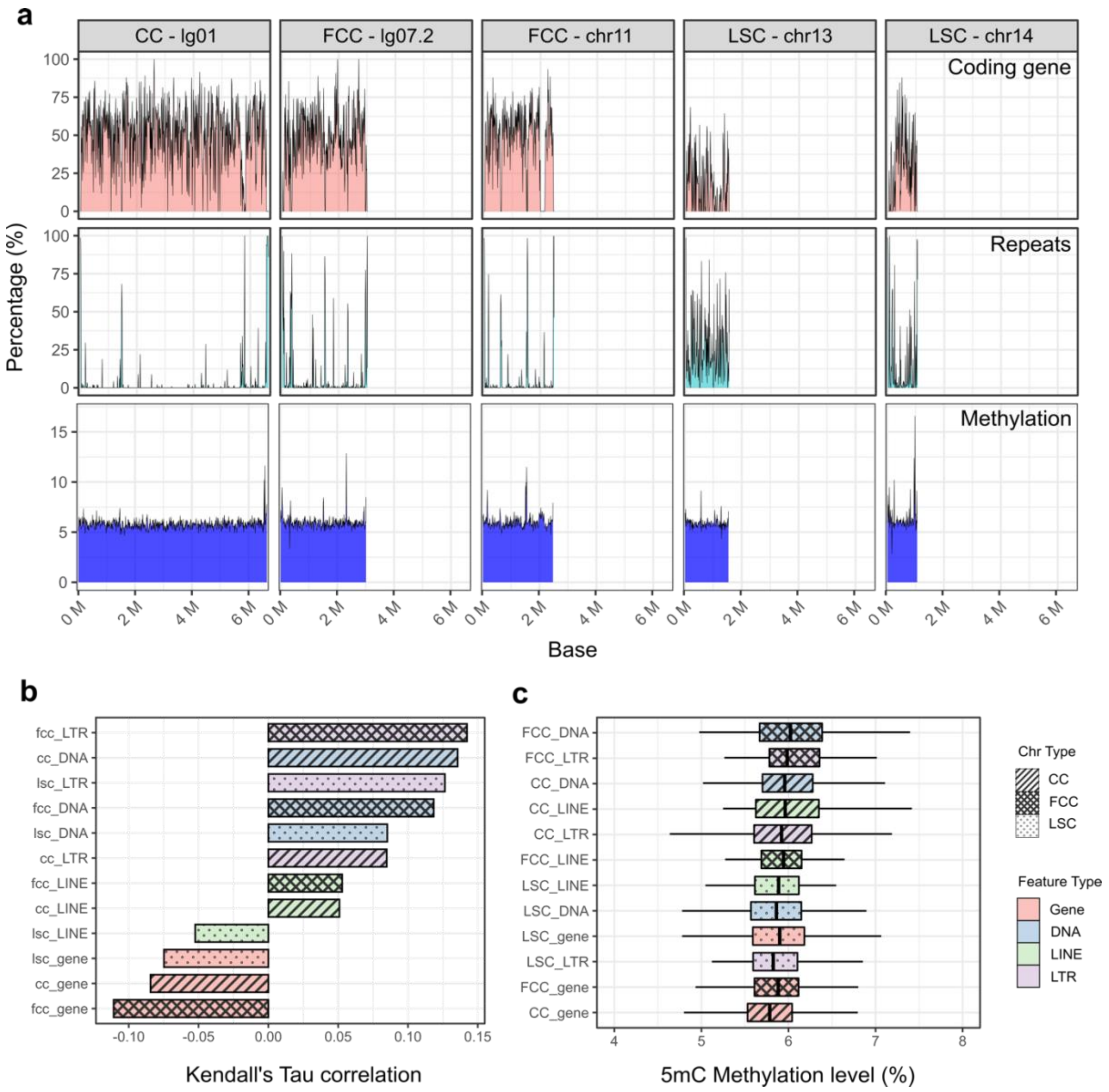

Fig. S13. Methylation analysis of *Fusarium* sp. Ph1. (a) Percentages of coding gene, repeats, and methylation across 10 kb window of chromosomes. (b) Pearson correlation coefficient between proportion of genome features and methylation. (c) Methylation level of genome features. CC, FCC, and LSC represents core chromosome, fast-core chromosome, and lineage-specific chromosome, respectively.

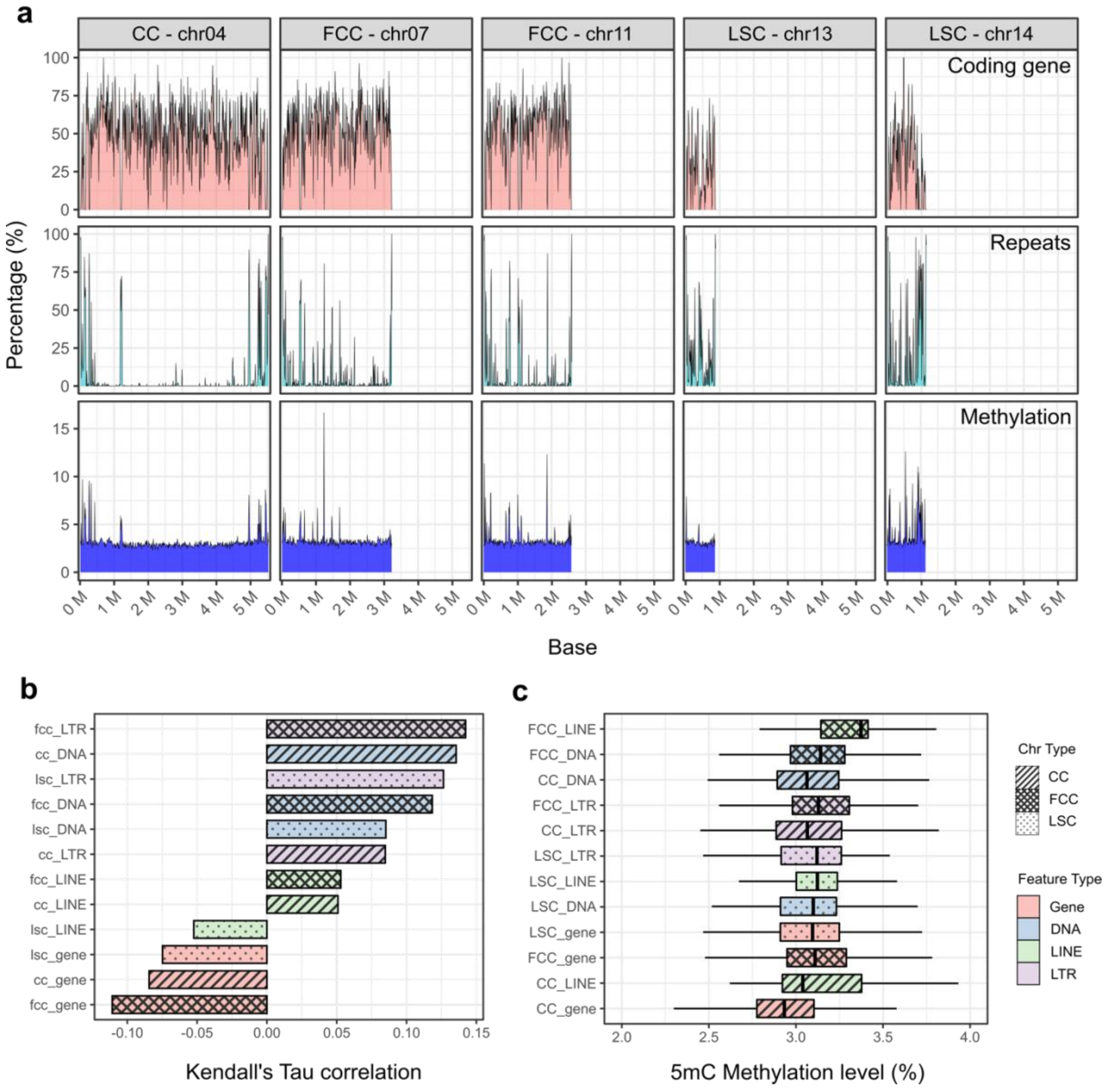

Fig. S14. Methylation analysis of *F. vanettenii* Fs6. (a) Percentages of coding gene, repeats, and methylation across 10 kb window of chromosomes. (b) Pearson correlation coefficient between proportion of genome features and methylation. (c) Methylation level of genome features. CC, FCC, and LSC represents core chromosome, fast-core chromosome, and lineage-specific chromosome, respectively.

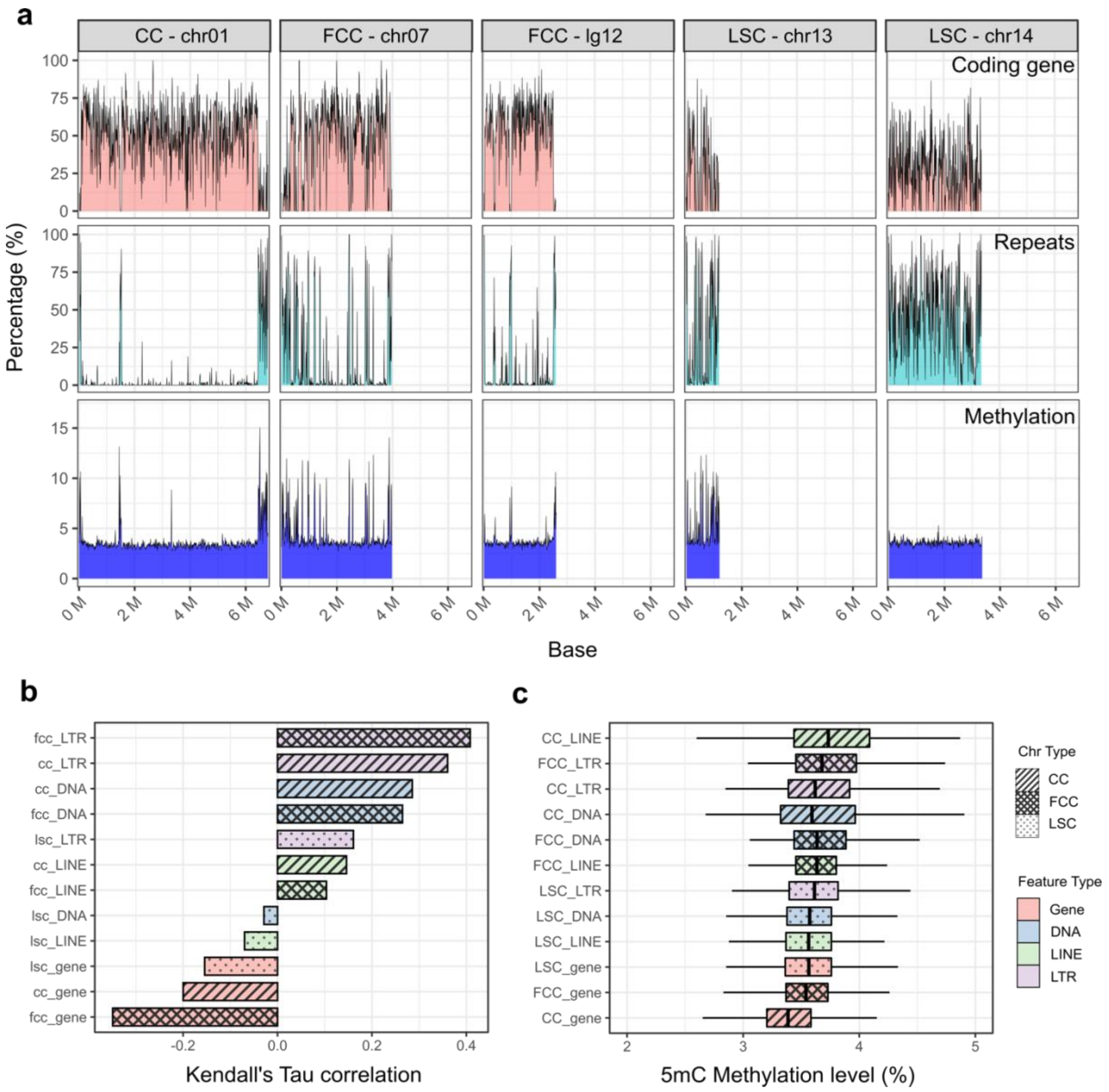

Fig. S15. Gene density in 10 kb window per chromosome or chromosome types. Plot were plotted using *F. falciforme* Fu3, *F. keratoplasticum* LHS11, *Fusarium* sp. Ph1, and *F. vanettenii* Fs6.

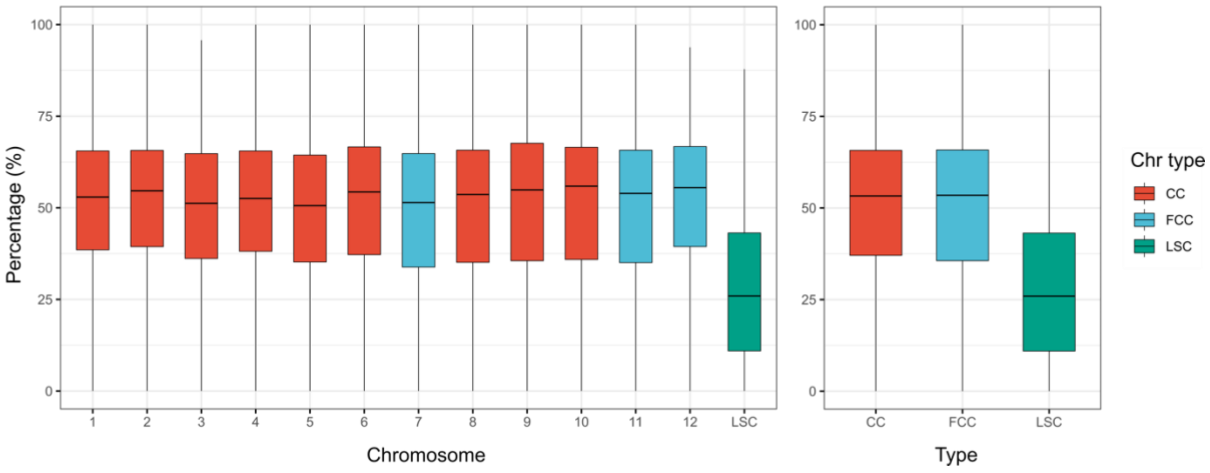

Fig. S16. Host attraction assay. (a) Experimental setup for control treatment using stopper (left tube) and experimental treatment using *P. sinensis* egg (right tube) placed horizontally upon beginning of experiment. (b) hyphae growth rate of all treatments had no significant difference tested using Wilcoxon-test (ns: $p > 0.05$) in each group comparisons.

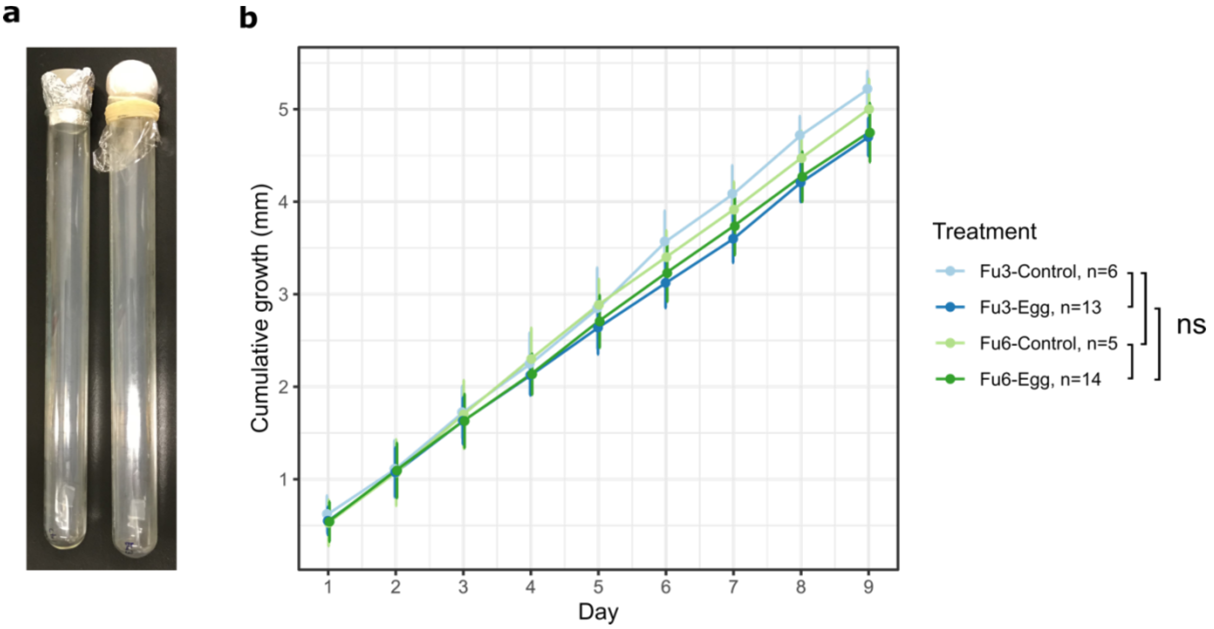

Fig. S17. Scanning electron microscopy images. Chinese soft-shelled turtle *Pelodiscus sinensis* eggshell inoculated with *F. falciforme* Fu3 or *F. keratoplasticum* Fu6 on five-dpi, showing fungal hyphae spreading on the eggshell surface. Arrowhead indicates hyphae growing into cavity-like structure.

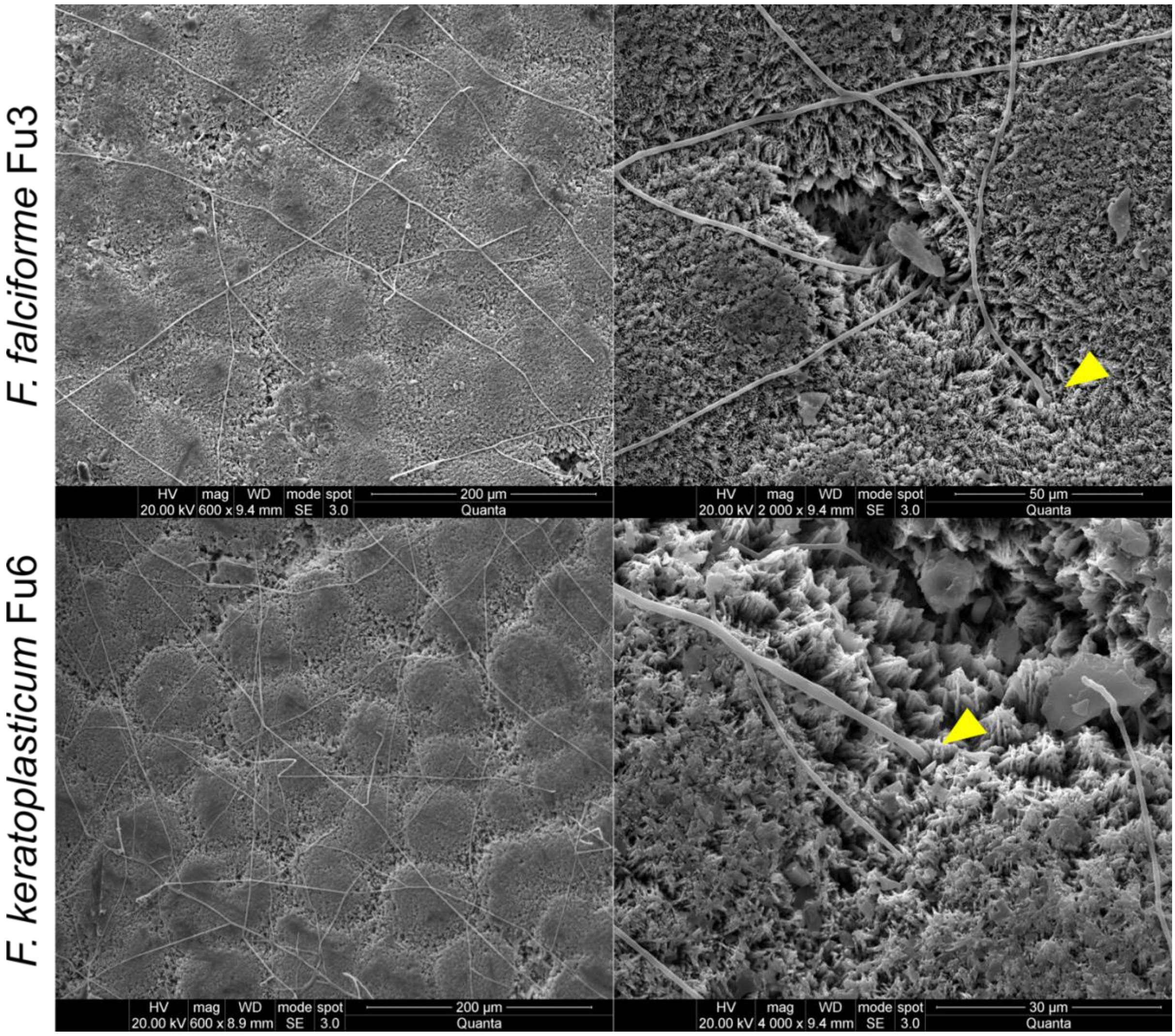

Fig. S18. Chinese soft-shelled turtle *P. sinensis* egg inoculated with conidia of *F. falciforme* Fu3 or *F. keratoplasticum* Fu6. (a) Fu6, four-dpi. (b) Fu3, three-dpi. (c) Fu6, three-dpi. Arrowhead indicates fungal colonization on (a) egg membrane as mycelium mass and (b and c) on the egg content as white blotches. Scale bar is approximately 5 mm.

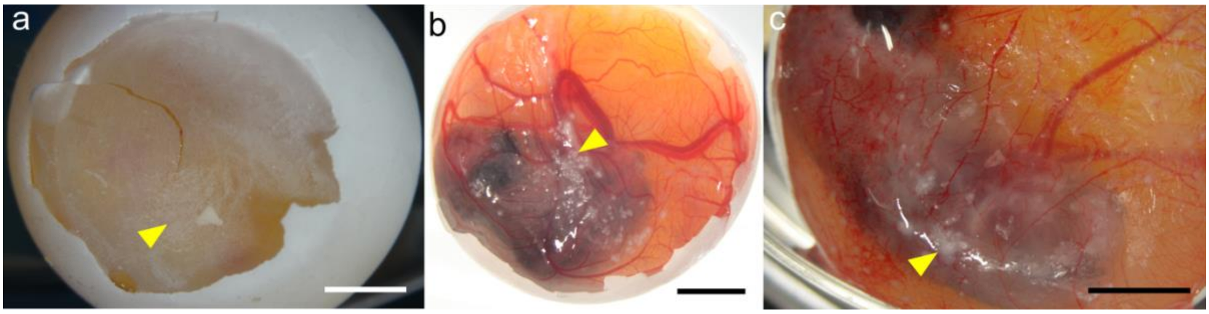

Fig. S19. Principal component analyses of pathogens' gene expression pattern of inoculated samples. (a) *F. falciforme* Fu3 (F) and (b) *F. keratoplasticum* Fu6 (K). "B" and "M" denote blotch and membrane samples, respectively.

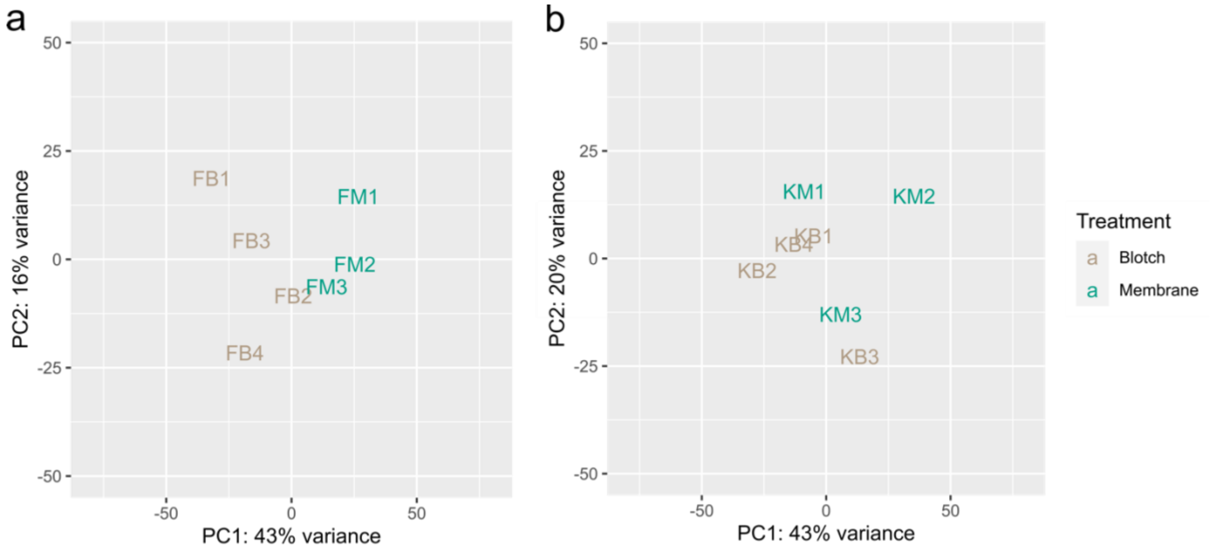

Fig. S20. Principal component analyses of pathogens' gene expression pattern of all samples. (a) *F. falciforme* Fu3 (F) and (b) *F. keratoplasticum* Fu6 (K). "C", "B" and "M" denote control sample, blotch and membrane of inoculated samples, respectively.

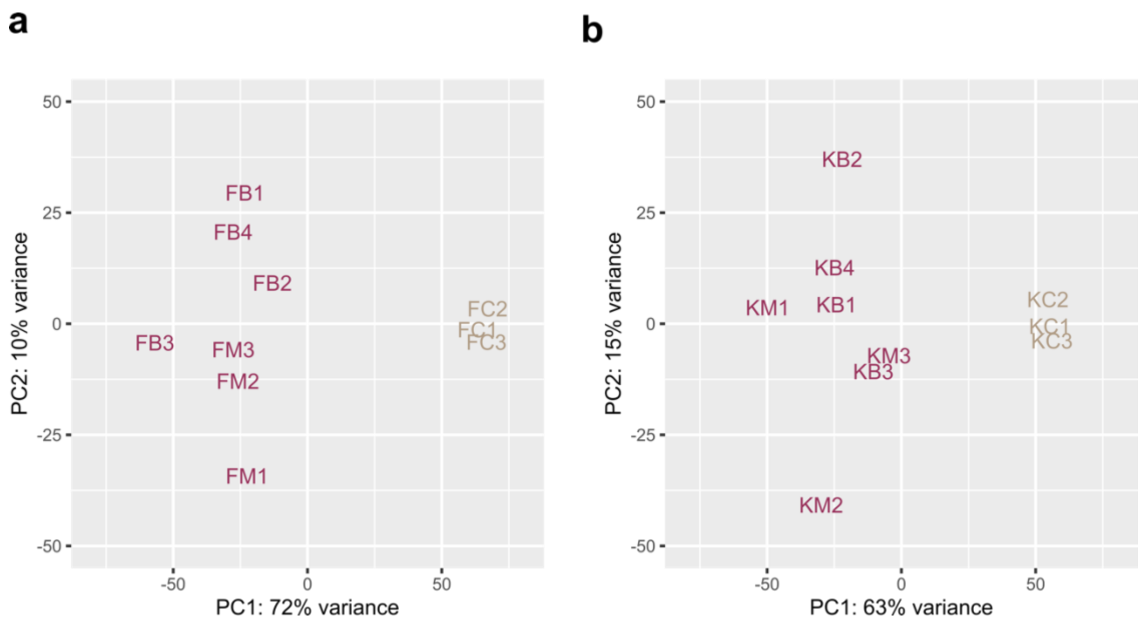

Fig. S21. Correlation plots of log2 normalized transcript per million (TPM) of one-to-one orthologous gene of *F. falciforme* Fu3 and *F. keratoplasticum* Fu6. (a) inoculated and (b) control (mycelium) samples.

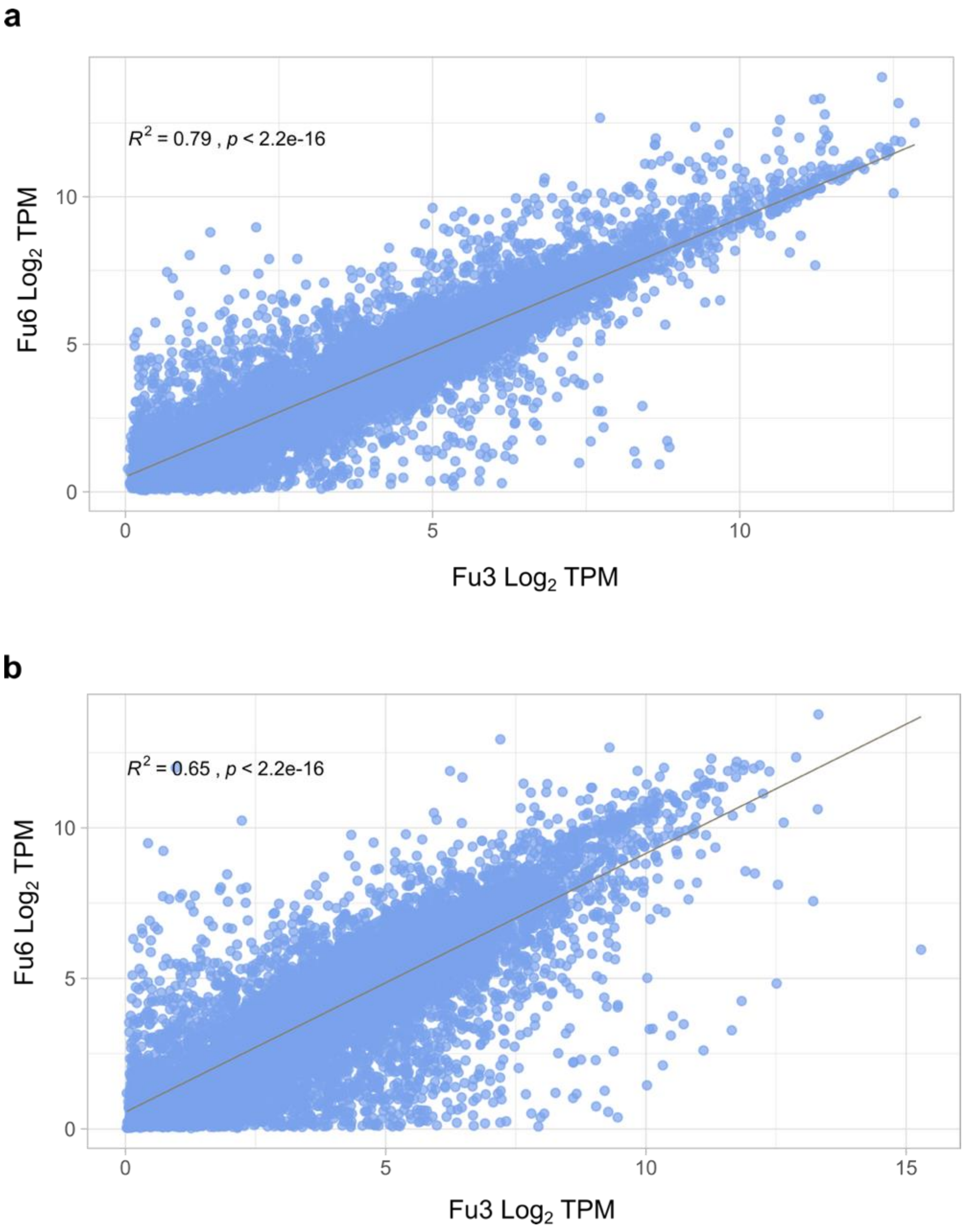

Fig. S22. Distribution of all differentially expressed genes of FSSC pathogens during egg inoculation experiment. (a) *F. falciforme* Fu3 and (b) *F. keratoplasticum* Fu6. Each red line represents single gene.

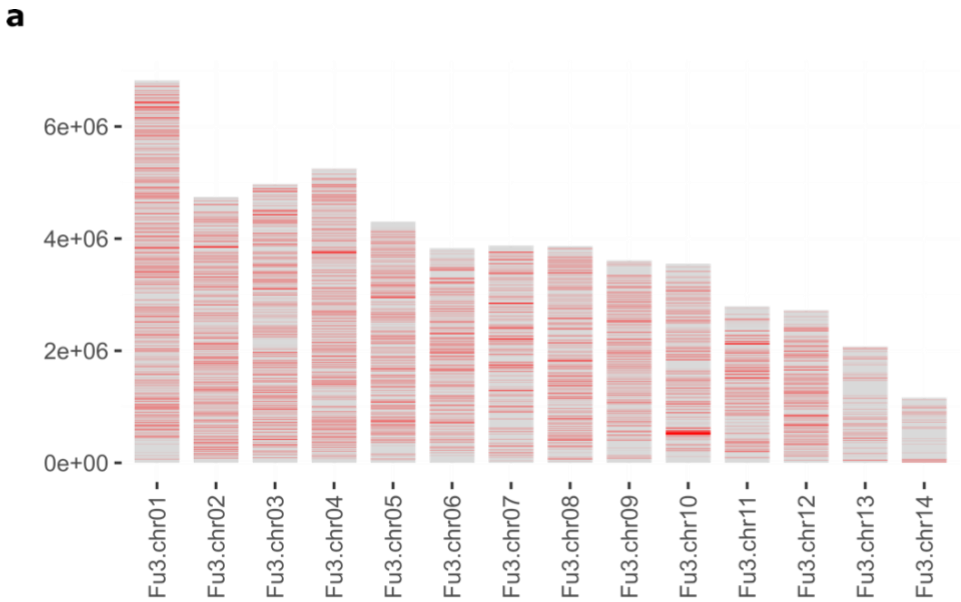

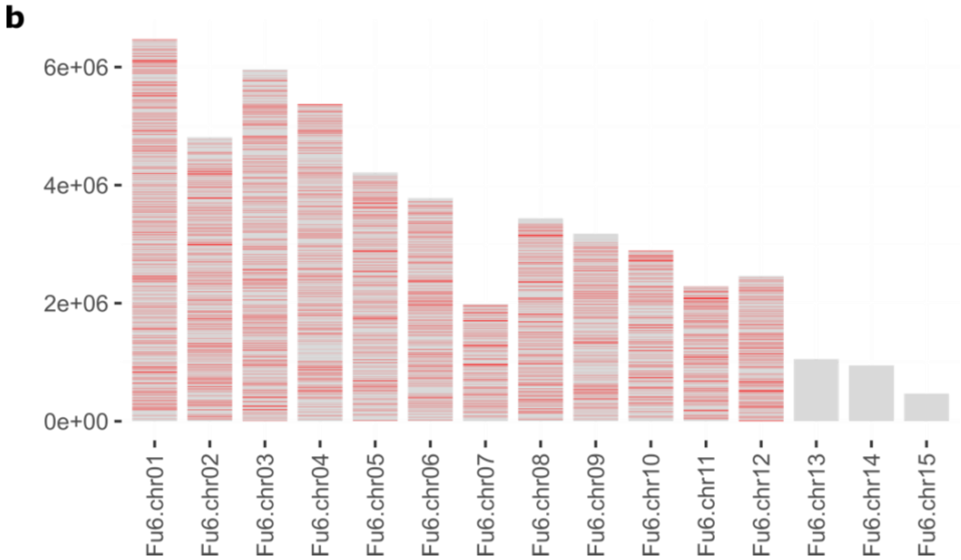

Fig. S23. Gene expression pattern of the animal host *P. sinensis*. (a) Principal component analyses (PCA) of gene expression pattern compared between host inoculated by *F. falciforme* Fu3 and *F. keratoplasticum* Fu6 (sample name starts with F and K, respectively) and natural developing host (DRR; Wang et al., 2013). (b) Correlation plot of log_2 normalized transcript per million (TPM) of host gene inoculated by *F. falciforme* Fu3 and *F. keratoplasticum* Fu6.

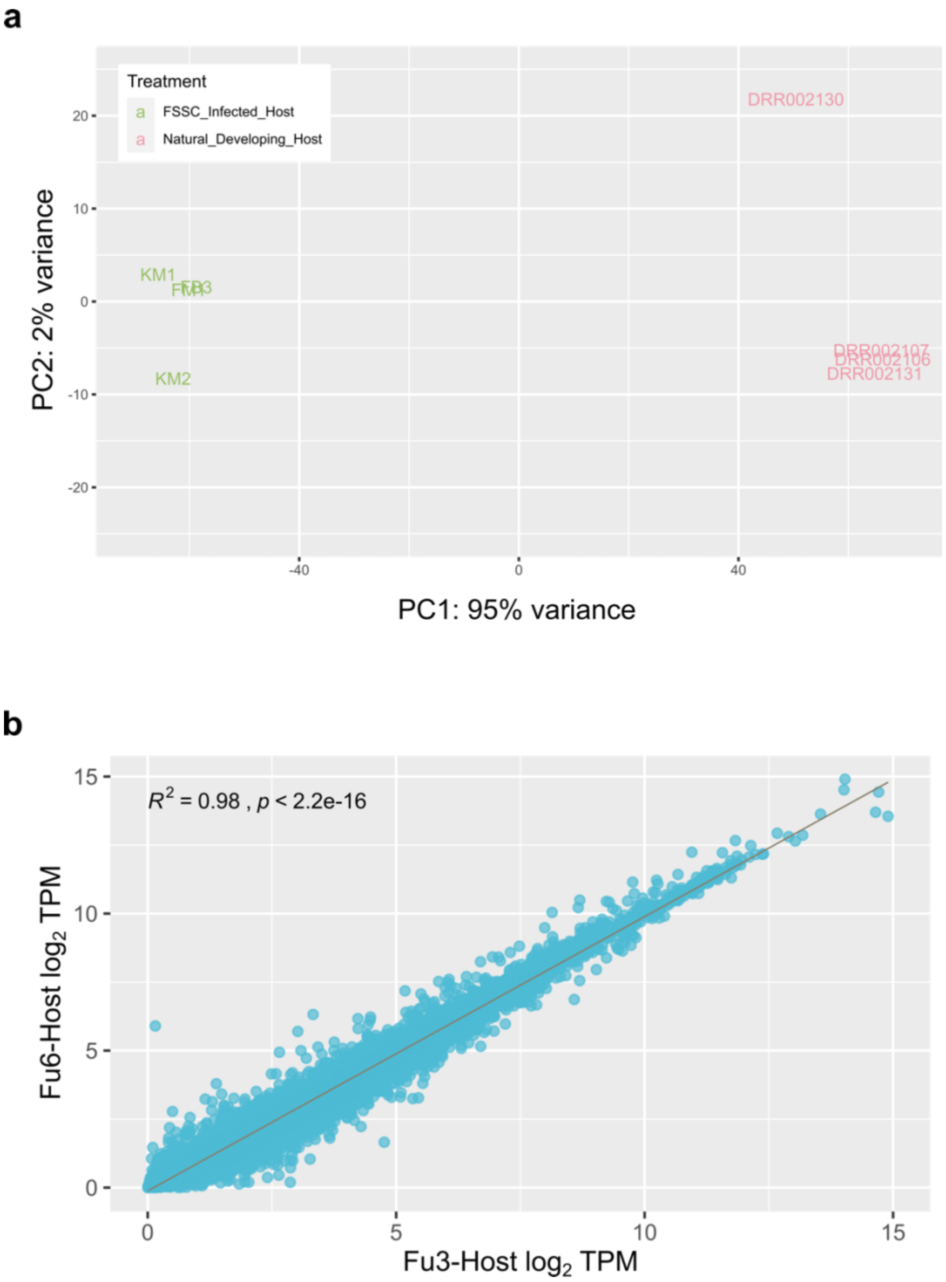

Supplement: Supplementary file 2 — Additional file 2: Fig. S1. Genome features in FSSC assemblies. Fig. S2. Multi-locus sequence typing (MLST) phylogeny tree of FSSC. Fig. S3. Genome phylogeny of Fusarium species. Fig. S4. Orthologue sharing amongst Fusarium chromosome. Fig. S5. FSSC genome synteny. Fig. S6. Synteny between F. vanettenii Fs6 and FVANE. Fig. S7. Location of FSSC-specific genes across genomes. Fig. S8. Selection level in FSSC chromosomes. Fig. S9. Orthogroup number comparison between F. falciforme Fu3 and F. oxysporum f. sp. lycopercisi 4287. Fig. S10. Syntenic dotplot between FSSC and non-FSSC species. Fig. S11. Methylation level of each chromosome in FSSC. Fig. S12. Methylation analysis of F. keratoplasticum LHS11. Fig. S13. Methylation analysis of Fusarium sp. Ph1. Fig. S14. Methylation analysis of F. vanettenii Fs6. Fig. S15. Gene density in 10 kb window per chromosome or chromosome types. Fig. S16. Host attraction assay. Fig. S17. Scanning electron microscopy images. Fig. S18. Chinese soft-shelled turtle P. sinensis egg inoculated with conidia of F. falciforme Fu3 or F. keratoplasticum Fu6. Fig. S19. Principal component analyses of pathogens’ gene expression pattern of inoculated samples. Fig. S20. Principal component analyses of pathogens’ gene expression pattern of all samples. Fig. S21. Correlation plots of log2 normalized transcript per million (TPM) of one-to-one orthologous gene of F. falciforme Fu3 and F. keratoplasticum Fu6. Fig. S22. Distribution of all differentially expressed genes of FSSC pathogens during egg inoculation experiment. Fig. S23. Gene expression pattern of the animal host P. sinensis. [file 12915_2022_1436_MOESM2_ESM.pdf]
