# Supplementary material for: Comparative genomic and transcriptomic analyses of trans-kingdom pathogen Fusarium solani species complex reveal degrees of compartmentalization
Source: BMC Biol. 2022 Oct 20;20:236. doi: 10.1186/s12915-022-01436-7 (PMC9583462; doi:10.1186/s12915-022-01436-7)
Supplement: Supplementary file 3 — Additional file 3. Extended Results and Methods. [file 12915_2022_1436_MOESM3_ESM.pdf]

# Additional File 3:
# Extended Result and Methods

for

# Comparative genomic and transcriptomic analyses of trans-kingdom pathogen *Fusarium solani* species complex reveal degrees of compartmentalization

Daphne Z. Hoh[1,2,3], Hsin-Han Lee[1], Naohisa Wada[1], Wei-An Liu[1], Min R. Lu[1], Cheng-Kuo Lai[1,4], Huei-Mien Ke[1], Pei-Feng Sun[1,2,3], Sen-Lin Tang[1,2], Wen-Hsin Chung[5], Ying-Lien Chen[6], Chia-Lin Chung[6] and Isheng Jason Tsai[*1,2,4]

*Corresponding author: Isheng Jason Tsai ijtsai@sinica.edu.tw

## Extended Results

**Fast-core and lineage-specific chromosomes are enriched in genes associated with pathogenicity and niche adaptation.**

While we expected different structural characteristics and selection pressures on each chromosome type, we asked whether these chromosome types have genes which are distinct in terms of biological functions. We first compared the mean proportion and number of effectors, carbohydrate-active enzymes and secondary metabolite biosynthetic genes clusters among the chromosome types and determined FCCs had the highest among all comparisons (Additional file 1: Table S11 and S12). The finding suggested FCCs were more likely to involve in pathogenicity processes during host colonization and infection. Annotation via Cluster of Orthologous Groups (COG) revealed the CCs had a higher proportion of genes related to biological functions associated with RNA processing and modification, nucleotide metabolism and transport, and translation, compared to FCCs and LSCs. Most of the genes in FCCs had COG category associated with metabolism and transport of carbohydrates and amino acids, energy production and conversion, secondary structure, defence mechanisms and biogenesis of cell wall, cell membrane and envelop. Lastly, LSCs had most genes in the COG category related to chromatin structure and dynamics, cell cycle control and mitosis, replication and repair, and transport and metabolism of inorganic ions.

We determined a significant overrepresentation of genes through Gene Ontology (GO) analysis in each chromosome type and found a similar pattern as in COG analysis. CCs contained enriched genes mainly involved in the core biological processes such as regulation, primary metabolism and biosynthesis of organic macromolecules (i.e., proteins and nucleic acids). FCCs had genes associated with processes such as hyphae and mycelium growth, cell wall biogenesis, response to host defences and stress, regulation of immune system processes, detoxification, secondary metabolites (i.e., toxin) biosynthesis, and metabolic process of carbohydrates, amino acids and ions. Finally, enriched genes in LSCs involve in the regulation of developmental processes such as cell differentiation, cell wall biogenesis, cell

morphogenesis, and asexual sporulation. LSCs also have genes involved in processes which respond to external stimulus and host, regulation of immune system processes, mycotoxin biosynthesis, and transmembrane transports of various kinds of materials which include ions, cation, and organic acids. In summary, FCCs and LSCs harbour genes which are feasibly linked to pathogenicity and expansion of new niches such as environment and new host, compared to CCs which mainly harbour genes associated with essential cellular functions.

**Gene expression pattern of FSSC during animal infection**

Principal component analysis shows that the expression pattern of single-copy orthologs in *F. falciforme* Fu3 and *F. keratoplasticum* Fu6 pathogens were separated by treatment types (Figure 6a; all samples in Additional file 2: Fig. S20), indicating both pathogen species responded distinctly after contacting an animal host compared to culture media. Significant high correlation was found in expression of single-copy orthologs in inoculated samples (Additional file 2: Fig. S21a; $Log_2$ TPM, $R^2 = 0.79$, $p < 2.2e^{-16}$) which was slightly higher compared to the control samples (Additional file 2: Fig. S21b; $Log_2$ TPM, $R^2 = 0.65$, $p < 2.2e^{-16}$), suggesting both species adopted similar colonization and infection strategy while contacting animal host.

## Extended Methods

### Fungal culturing conditions

Six isolates from *Fusarium solani* species complex (FSSC) clade 3 of this study – *Fusarium falciforme* (Fu3), *F. keratoplasticum* (Fu6), *F. keratoplasticum* (LHS11), *Fusarium* sp. haplotype FSSC12 (LHS14), *Fusarium* sp. (Ph1), and *F. vanettenii* (Fs6) underwent the same culture conditions for gDNA and RNA extractions. The isolates were cultured on 1/2 potato dextrose agar (PDA) at 28°C in dark for seven days. *F. falciforme* Fu3 and *F. keratoplasticum* Fu6, which were previously isolated from dead sea turtle eggs, were used to conduct additional pathological experiments that include host attraction assay, animal inoculations and histological observations during disease establishment. For host attraction assay, *F. falciforme* Fu3 and *F. keratoplasticum* Fu6 were cultured on 1.5% water agar at 28°C in dark for five days. *F. falciforme* Fu3 and *F. keratoplasticum* Fu6 cultures prepared for animal inoculations and histological observations experiments were cultured on 1/2 PDA at 28°C in dark for seven days.

### Species identification of isolates

DNA isolation of FSSC mycelium cultures was carried out using ZYMO Quick-DNA Fungal/Bacterial Miniprep Kit (ZYMO Research, Irvine, USA, Cat. #D6005). The identity of these isolates was determined via multi-locus sequence typing (MLST) of ITS rDNA (ITS5), RPB2 (7cF/11aR), and TEF1 (EF1/EF2) regions (O'Donnell et al., 2008), incorporating other FSSC sequences which species identity were determined (Additional file 1: Table S9). PCR conditions for all primers followed Liu et al., (1999) and phylogenetic analysis was performed as described in Hoh et al., (2020).

### Assignment of FSSC linkage groups

The FSSC linkage groups were assigned by aligning the *F. falciforme* Fu3 assembly against the published *F. vanettenii* 77-13-4 genome (Coleman et al., 2009) using MUMmer4 (Marçais et al., 2018). The *F. falciforme* Fu3 genome was then used as the reference for alignment of the other five FSSC genomes. Additional evidences were checked by clustering of *F. falciforme* Fu3 and other FSSC genomes via one-to-

one orthologous gene (Additional file 2: Fig. S5). We defined synteny as the conserved region between the genomes in comparison based on one-to-one gene, in which the cluster of genes was colinear and located on the same chromosome or linkage group. We designated the contigs of all FSSC assemblies based on: (1) contigs which were completely assembled (telomere-to-telomere) were named “chromosome”, abbreviated in figures as “Chr”; (2) incomplete contigs which were largely syntenic with other FSSC genomes were named “linkage group”, abbreviated in figures as “LG”; and (3) incomplete contigs which were conserved with other FSSC genomes were named as “contig”, abbreviated as “cont”. In addition, we used PROmer (v3.23; Kurtz et al., 2004) to determine synteny between FSSC and non-FSSC species.

**Animal sample preparation**

Freshly laid and fertilized soft-shelled turtle (*Pelodiscus sinensis*) eggs were purchased from a local farm in Pingtung, Taiwan. The eggs were embedded in styrene foam to avoid movement-induced mortality during transportation. The top of the egg surface was marked with a pencil to ensure eggs were not rotated during the following process: egg surface was cleaned with a brush to remove dirt and surface-sterilized by immersing in 75% EtOH for 1 min, 1% bleach + Tween 20 solution for 1 min, and autoclaved distilled water for 1 min. Egg surface was wiped dry using clean tissue paper and half-buried in sterilized and moist vermiculite (1g water/1g vermiculite) in a plastic container covered with cling film. Each container contained ten eggs and was incubated in a climate chamber (Panasonic MLR-352H, Gunma, Japan) at 28°C and 60% relative humidity in the dark until the 30$^{th}$ day. On day 30, the eggs were observed using the candling technique to check for embryo viability. These embryos were estimated to be developed to stage TK21 to 22 (Tokita & Kuratani, 2001). Dead eggs were removed and alive eggs were kept for the following experiments which included pathogen inoculation, host attraction assay and eggshell observations.

**Attraction assays**

To determine if the pathogens *F. falciforme* Fu3 and *F. keratoplasticum* Fu6 can be attracted to the egg host, an attraction assay experiment was performed in glass

tubes (10cm tube length and 2cm diameter) placed horizontally and filled with approximately 15mL of 1.5% water agar. An approximately 0.5cm$^3$ mycelial block was cut from the margin of a 1.5% water agar and carefully transferred to the end of the glass tube without touching the tube surface and bottom agar. At the opening of the glass tube, the egg was fixed using cling film and rubber band in the experimental group while a stopper was used in the control group (Additional file 2: Fig. S16a). The horizontally placed tubes were incubated at 28°C in dark and hyphal growth was recorded every day for nine days. The experiment was repeated twice with a total of 38 samples (tubes) assessed.

**Histology during initial disease establishment**

*P. sinensis* eggs were inoculated by placing an approximately 1cm$^3$ mycelial block (cut from the margin of the colony) on the shell surface and incubated at 28°C in the dark. After five days, the mycelial block was removed and eggshell fragments surrounding the mycelial block were cut and collected for scanning electron microscopy (SEM) and laser scanning confocal microscopy (LSCM) observations. For SEM, eggshell fragments were first fixed with 4% paraformaldehyde and 2.5% glutaraldehyde in 1x PBS buffer for 1 hour at 4°C. Samples were then washed with 1x PBS buffer thrice for 10 min each, followed by second fixation using 1% osmium tetroxide-buffered solution for 1 hour in the dark at room temperature. Fixed samples were washed again as previously described and went through a serial dehydration step using EtOH at 30, 50, 70, 80, 90, 95, and 100% concentration for 10 minutes at each step. Dehydration using 100% EtOH was repeated twice and samples were then dried in Pelco CPD#2400 $CO_2$ critical point dryer (Ted Pella Inc., Redding, USA). Finally, samples were coated with a layer of gold with Sputter Coater 108 auto (Cressington Scientific Instruments, Watford, UK) and examined using Quanta 200 ESEM (FEI Company, Hilsboro, USA). For LSCM, eggshells fragments were placed into warm 1.5% agarose and waited until solidified. Each solidified sample embedded in agarose was transferred to a 50mL Falcon tube and fixed with 10% formalin overnight at room temperature. Fixed samples were washed with PBS buffer thrice and then kept in PBS buffer for storage at 4°C until further processing. Sample embedding and undecalcified sectioning procedures

followed Wada et al. (2016). Each section was cut into 8µm thickness using an adhesive film and a tungsten carbide blade (SL-T25, Section-Lab Co. Ltd, Japan) on Leica CM3050 cryostat (Leica Microsystems, Nussloch, Germany). The sections on the adhesive film were directly stained with Calcofluor White Stain (Sigma-Aldrich, #18909) for a few minutes, washed with water to remove excess stain, and mounted with ProLong™ Gold Antifade Mountant (Thermo Fisher Scientific, Waltham, MA, USA, #P36930), followed by removing excess mounting solution and covered with a coverslip. Slides were kept at 4°C in the dark until LSCM observation. Samples were examined using LSM880 (ZEISS, Germany) and visualized with Zen2.3 software black edition (ZEISS, Germany). Signals of Calcofluor White were excited with 405nm laser and detected in range 410–523nm. DIC (differential interference contrast) images were also acquisted. All image acquisitions were scanned with a 40x objective lens in z-stack mode (0.391µm). The z-stack images were prepared with a maximum intensity projection through Zen2.3 software lite edition (ZEISS, Germany).

**Animal inoculation with FSSC for transcriptome profiling**

During candling observation, the location of the embryo and directly opposite to the embryo (upper pole) were marked. The observed and alive eggs were half-buried on fresh sterilized and moist vermiculite with embryos placed at the lower pole (buried). A tiny hole was carefully made on top of the eggshell and this created a small air space under the hole and above the egg content. Spore suspension of the cultures was prepared by washing the mycelium with 1x PBS solution and filtered through 40µm cell strainer. Haemocytometer-estimated $10^7$ spores/mL suspension was injected into the egg's air space through the tiny hole without poking the egg content. The inoculated eggs were incubated at 28°C and 60% relative humidity in the dark for another three to four days. The mycelium of *F. falciforme* Fu3 and *F. keratoplasticum* Fu6 collected from 4-day-old colony grown on 1/2 PDA were used as the control. At 3 to 4 dpi, eggs were made broken to check the embryo's vitality (Additional file 1: Table S26) and samples were collected for RNA sequencing. This timepoint was chosen under the assumption of sufficient time for fungi to start and carry out the infection. In addition, while host was identified as alive during pathogen inoculation, we tried to avoid the possibility of

decomposing dead host at earlier post-inoculation (1 to 2 dpi) to affect the outcome of gene expression. Samples collected for RNA isolation are of two types depending on the fungal growth during collection: (1) the presence of mycelium mass as white blotches on the egg content (such as embryo and yolk) and (2) cotton-wool-like growth on the opaque eggshell membrane (Additional file 2: Fig. S18). Since this experiment focused on the pathogen, we tried to collect only the white substances as much as it is available and the shell membrane with obvious fungal growth. Each sample type was considered one inoculated sample and was named “B” and “M” representing “blotch” and “membrane” respectively. Samples were collected using sterilized forceps and kept immediately in TRIzol reagent (Thermo Fisher Scientific, Waltham, MA, USA, Cat. #15596026) and –80°C until further processing.
